# Agent-based vs. equation-based multi-scale modeling for macrophage polarization

**DOI:** 10.1371/journal.pone.0270779

**Published:** 2024-01-25

**Authors:** Sarah B. Minucci, Rebecca L. Heise, Angela M. Reynolds

**Affiliations:** 1 Department of Mathematics & Applied Mathematics, Virginia Commonwealth University, Richmond, VA, United States of America; 2 Department of Biomedical Engineering, Virginia Commonwealth University, Richmond, VA, United States of America; INSERM, FRANCE

## Abstract

Macrophages show high plasticity and result in heterogenic subpopulations or polarized states identified by specific cellular markers. These immune cells are typically characterized as pro-inflammatory, or classically activated M1, and anti-inflammatory, or alternatively activated M2. However, a more precise definition places them along a spectrum of activation where they may exhibit a number of pro- or anti-inflammatory roles. To understand M1-M2 dynamics in the context of a localized response and explore the results of different mathematical modeling approaches based on the same biology, we utilized two different modeling techniques, ordinary differential equation (ODE) modeling and agent-based modeling (ABM), to simulate the spectrum of macrophage activation to general pro- and anti-inflammatory stimuli on an individual and multi-cell level. The ODE model includes two hallmark pro- and anti-inflammatory signaling pathways and the ABM incorporates similar M1-M2 dynamics but in a spatio-temporal platform. Both models link molecular signaling with cellular-level dynamics. We then performed simulations with various initial conditions to replicate different experimental setups. Similar results were observed in both models after tuning to a common calibrating experiment. Comparing the two models’ results sheds light on the important features of each modeling approach. When more data is available these features can be considered when choosing techniques to best fit the needs of the modeler and application.

## Introduction

Macrophage polarization refers to the approximate state of activation of a macrophage responding to its environment. Macrophages show high plasticity and result in heterogenic subpopulations or polarized states identified by specific cellular markers [1]. Macrophage phenotypes may be largely classified as either pro-inflammatory or pro-injurious, also called classical macrophage polarization, or they can reflect an alternative activation profile, which has been considered as anti-inflammatory or pro-repair [2–4]. Classically activated (M1) macrophages promote the development of acute injury, whereas alternatively-activated macrophages (M2) may be involved in limiting or resolving inflammation [1]. Macrophage polarization is highly involved in physiological transitions from inflammation to tissue regeneration. A major field of macrophage biology seeks to understand the mechanisms and pathways leading to macrophage polarization. Macrophage polarization depends heavily on the tissue microenvironment and disease or injury state in which the cells are responding. Computational models provide an avenue to examine the many variables leading to macrophage polarization states.

The plasticity of macrophages has a significant impact on the overall ability of the immune system to resolve the insult [5]. Several mathematical models have been published that include macrophage polarization, including ODE models of subcellular signaling and simplified M1/M2 activation. Maiti et al. [6] and Moya et al. [7] focused on the subcellular signaling pathways of NF-*κ*B/TNF*α* and STAT3/IL-10, respectively. Frank et al. [8] and Zhao et al. [9] developed two-dimensional ODE models with M1 and M2 activation as the state variables. Rex et al. [10] used a Boolean model to select genes related to M1/M2 dynamics and developed an ODE modeling the dynamics of those genes. Additionally, some modeling efforts of macrophage plasticity incorporate spatial dynamics. Agent-based models that include M1/M2 phenotypes have been developed in the context of tuberculosis Kirschner et al. [11] and Nickaeen et al. [12] developed a PDE model of M1/M2 macrophages in response to high levels of IL4 or LPS/IFN*γ*.

In this work, we propose two models of the immune response to general inflammation that build upon previous models to examine the spectrum of macrophage activation in greater detail and guide future modeling and experimental efforts by comparing different approaches [3, 13, 14]. We do not attempt to capture the behavior within an entire organ; rather, we model a response with a small number of cells to understand how local immune signaling affects macrophage activation. This model is an extension of work by Maiti et al. [6]; we added details of the IL-10 pathway not yet included in Maiti et al., specifically additional interactions between the pro- and anti-inflammatory components, a more detailed IL-10 signaling cascade, as well as feedback loops to resolve the anti-inflammatory response, by adapting and extending equations from Moya et al. [7]. The model consists of a small number of macrophages, each of which has a set of equations modeling its subcellular pathways. These macrophages are linked by external TNF*α* and IL-10, which can be both introduced into the system at various times and produced by the macrophages themselves. In our ABM, we incorporated pro- and anti-inflammatory mediators, allowed for M1/M2 activation to occur on a spectrum, and accounted for spatial dynamics. In this model, macrophages can become more activated towards an M1 or M2 phenotype based on their local patch environment, and perform a variety of roles depending on their activation levels. Both models account for macrophage cell cycle using randomly generated lifespans for each macrophage.

Based on data from Maiti et al. [6], we calibrated the models to each other by simulating a single macrophage with both pro- and anti-inflammatory stimuli. Through this initial scenario, we found that modeling the SOCS regulatory feedback loop is important in the definitive resolution of inflammation. We then simulated additional scenarios highlighting the effects of incorporating cell lifespan, recruitment, and various types of external stimuli and initial conditions. Comparison of these scenarios between the ODE model and ABM revealed overall similar behavior of M1 and M2 activation across two very different modeling approaches, suggesting that detailed subcellular pathway modeling is not necessary to achieve complex interplay between M1 and M2 polarization.

In the following sections, we describe the models in detail, the calibrating experiment, and the comparison of various simulated scenarios.

## Methods

### ODE subcellular macrophage model

#### Biological summary

There are several main interactions involved in cell signaling pathways that we include in our model. First, extracellular signals such as TNF*α* and IL-10 bind to and unbind from their receptors on the cell surface. Receptors transmit signals to other proteins within the cell, which may become activated or phosphorylated [15]. These complexes induce activation of transcription factors, proteins that are responsible for translocating to the nucleus, where they control the transcription of specific genes in the DNA into mRNA. mRNA then undergoes translation in the cytosol, where the protein corresponding to the gene is assembled according to the mRNA sequence [16]. We also account for degradation of various components. We model this process using the law of mass action unless otherwise specified. Details for these interactions are given in the following sections.

TNF*α* triggers a signaling pathway that leads to activation of the transcription factor NF*κ*B and the subsequent shift to an M1 phenotype [17]. This results in the production of additional TNF*α* and IL-10 as well as other proteins. Alternatively, IL-10 activates the transcription factor STAT3 through the Jak-STAT pathway, giving rise to M2-type activation [18]. To capture the interactions between these pathways, we developed an ODE model, adapted from Maiti et al. [6] that includes these hallmark signaling pathways. This involves subcellular interactions between receptors and proteins in the cytosol and nucleus of the macrophage.

The model by Maiti et al. [6] initiates their signaling cascade with LPS, a molecule found in Gram-negative bacteria used to experimentally induce an immune response. IKK, a protein whose role is to regulate phosphorylation of I*κ*B*α*, is activated by both LPS and TNF*α*. Since we model general lung inflammation rather than a bacterial infection, we do not rely on activation of the M1 pathway by LPS. TNF*α* is a cytokine that is upregulated in inflammatory responses and involved in the inflammatory cascade, including triggering NF*κ*B transcription. Maiti et al. [6] include production of IL-10 and STAT3; our model extends this by including additional components of the Jak-STAT pathway and the negative feedback loops required to resolve the immune response. In the following sections, we note specifically which equations and terms are novel to our model.

Extracellular TNF*α* binds to its receptor, activating neutral IKK. IKK then phosphorylates I*κ*B*α* in the I*κ*B*α*-NF*κ*B complex, freeing NF*κ*B to translocate to the nucleus. In the absence of a stimulus, I*κ*B*α* sequesters NF*κ*B to prevent it from causing the production of unnecessary proteins. Transcription factor NF*κ*B initiates transcription of TNF*α*, IL-10, A20, and I*κ*B*α* mRNA, resulting in their translation and protein production. As part of a negative feedback loop that prevents excessive production of these proteins, A20 inactivates active IKK and I*κ*B*α* sequesters unbound NF*κ*B. TNF*α* and IL-10 are secreted from the cell.

IL-10 binds to its receptor, and JAK and Tyk tyrosine kinases, whose main function is to activate STAT3, bind to this complex as well. Without all of these components, STAT3 cannot be phosphorylated and control transcription of key genes in the nucleus. The IL-10-Jak-Tyk complex activates STAT3, which translocates to the nucleus and initiates the production of IL-10, SOCS1, and SOCS3. Both SOCS1 and SOCS3 are part of negative feedback loops that bring about resolution of both the M1 and M2 pathways. SOCS3 inhibits transcription of TNF*α* mRNA and both SOCS1 and SOCS3 inhibit activation of STAT3. IL-10 also inhibits activation of IKK.

**Fig 1:**
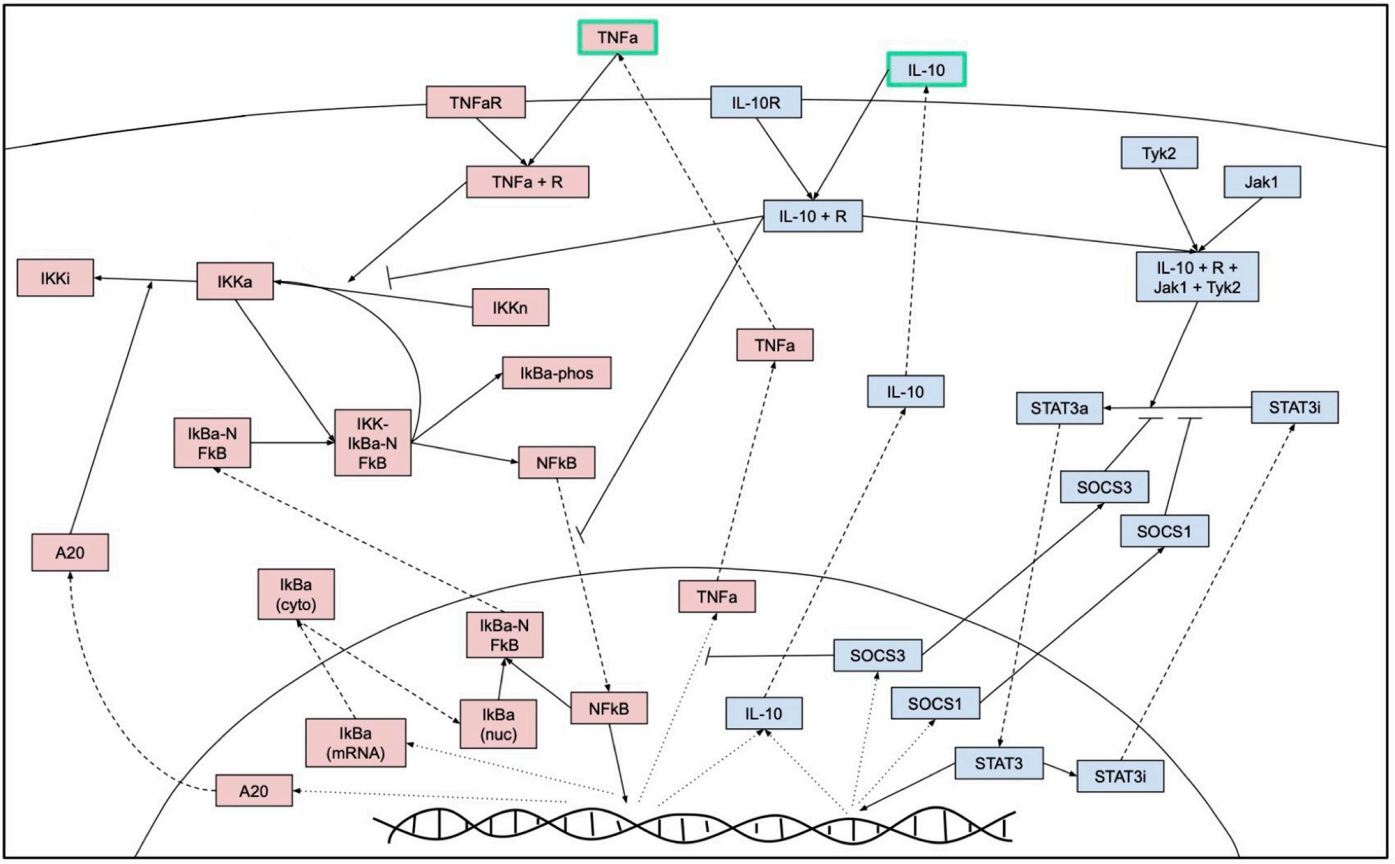
Model schematic. Schematic of interactions within a macrophage and with external stimuli TNF*α* and IL-10. Dashed lines represent interactions that involve movement between the cytosol and nucleus, dotted lines represent transcription processes in the nucelus, and solid lines represent all other interactions. Red boxes represent components that are primarily associated with the pro-inflammatory/M1 pathway and blue boxes with the anti-inflammatory/M2 pathway. Abbreviations: TNFa: tumor necrosis factor alpha, TNFaR: TNFa receptor, IL10: interleukin-10, IL10R: IL10 receptor, IKKi/a/n: inactive/active/neutral I*κ*B kinase, NFkB: nuclear factor *κ*B, IkBa-phos: phosphorylated I*κ*B*α*, IkBa (cyto/nuclear): I*κ*B*α* in cytosol/nucleus, Tyk2: tyrosine kinase 2, Jak1: Janus kinase 1, STAT3a/i: active/inactive signal transducer and activator of transcription 3, SOCS: suppressor of cytokine signaling.

Fig 1 summarizes these interactions, described in more detail in the equations. The schematic describes interactions between receptors, transcription factors, and other proteins within the cell in response to detection of extracellular signals on the cell surface. Table 1 lists the parameters used in the model and their descriptions. In this section we describe the changes and additions made to the model by Maiti et al. [6]. The full set of equations and corresponding code can be found in the S1 File.

**Table 1:** List of final parameter values for the subcellular pathways ODE model.

	Parameter	Description	Value	Unit
1.	*a*_*trans*_	Rate at which *A*20 is translated by *NFκB*	11.297	1/s
2.	*c*_*tf*_	Maximum *NFκB* concentration in nucleus	0.115	*μ*M
3.	*c*_*tfstat*3_	Maximum *STAT*3 concentration in nucleus	0.0714	*μ*M
4.	*e*_*ki*_	Rate at which *IκBα* is imported outside nucleus	3.570×10^−4^	1/s
5.	*e*_*ni*_	Rate at which *IκBα*-NF*κ*B is exported outside nucleus	0.158	1/s
6.	*i*_*ki*_	Rate at which *IκBα* is imported into nucleus	0.0154	1/s
7.	*il*10_*max*_	*IL*10/*IL*10*R* maximum concentration	3.003×10^−5^	*μ*M
8.	*i*_*ln*_	Rate at which *NFκB* is imported into the nucleus	0.002	1/s
9.	*k*_*bal*_	Component balance for TNF*α* and IL-10	0.0018	1/s
10.	*k*_*ikbatrans*_	Rate at which *IκBα* is translated by *NFκB*	0.180	1/s
11.	*k*_*dega*20_	Rate at which *A*20 decays	5.719×10^−4^	1/s
12.	*k*_*degikba*_	Rate at which phosphorylated *IκBα* decays	2.108×10^−4^	1/s
13.	*k*_*degtnfa*_	Rate at which extracellular *TNFα* is degraded	5.953×10^−4^	1/s
14.	*k*_*f*3_	Rate at which *TNFα* binds to its receptor	0.040	1/(*μ*M⋅s)
15.	*k*_*f*4_	Rate at which *IκBα* and *NFκB* associate	0.0013	1/(*μ*M⋅s)
16.	*k*_*fi*_	Rate at which *IKK* is activated	0.092	1/s
17.	*k*_*ilc*_	Rate at which *IL*10_*cyto*_ moves outside the cell	1.636×10^−4^	1/s
18.	*k*_*iljb*_	Rate at which *JAK*1 and *Tyk*2 are recruited to the *IL*10 complex	0.0076	1/(*μ*M^2^⋅s)
19.	*k*_*ilju*_	Rate at which *JAK*1 and *Tyk*2 unbind from the *IL*10 complex	0.0246	1/s
20.	*k*_*ilm*_	Rate at which *IL*_*mRNA*_ move from the nucleus to the cytosol	0.342	1/s
21.	*k*_*ilnf*_	Rate at which *IL*10_*mRNA*_ is transcribed by *NFκB*	0.229	1/s
22.	*k*_*ilrb*_	Rate at which *IL*10_*ext*_ binds to its receptor	0.007	1/(*μ*M⋅s)
23.	*k*_*ilru*_	Rate at which *IL*10_*ext*_ unbinds from its receptor	4.418×10^−4^	1/s
24.	*k*_*ilsn*_	Rate at which *IL*10_*mRNA*_ is transcribed by *STAT*3	0.938	1/s
25.	*k*_*in*_	Inhibition by IL-10: $\text{max}(1-\frac{IL10/R}{IL10/R_{max}},0)$	Varies	
26.	*k*_*k*1_	Rate at which *IKK* is inactivated by *A*20	0.0336	1/(*μ*M⋅s)
27.	*k*_*k*3_	Rate at which *IKK* associates with *IκBα*−*NFκB*	0.936	1/(*μ*M⋅s)
28.	*k*_*r*3_	Rate at which *TNFα* dissociates from its receptor	0.0031	1/s
29.	*k*_*s*1_	Rate at which *SOCS*1_*mRNA*_ moves into the cytosol	1.0192	1/s
30.	*k*_*s*1*st*_	Rate at which *SOCS*1_*mRNA*_ is transcribed by *STAT*3	1.970	1/s
31.	*k*_*s*3_	Rate at which *SOCS*3_*mRNA*_ moves into the cytosol	0.0047	1/s
32.	*k*_*s*3*st*_	Rate at which *SOCS*3_*mRNA*_ is transcribed by *STAT*3	2.701	1/s
33.	*k*_*sa*_	Rate at which activated STAT3 moves into nucleus	6.939×10^−5^	1/s
34.	*k*_*sec*_	Rate at which *TNFα* is secreted from the cytosol outside the cell	1.427×10^−4^	1/s
35.	*k*_*sni*_	Rate at which activated *STAT*3 in the nucleus becomes deactivated	9.532×10^−5^	1/s
36.	*k*_*snicyto*_	Rate at which inactivated *STAT*3 in the nucleus moves into the cytosol	0.0081	1/s
37.	*k*_*stat*_	Rate at which IL-10 complex activates STAT3	0.0089	1/(*μ*M^2^⋅s)
38.	*k*_*tnfatrans*_	Rate at which *TNFα* is translated by *NFκB*	0.388	1/s
39.	*k*_*v*_	Nuclear:cytoplasmic ratio (volume)	1.045	1/s
40.	*μ*_*a*20*m*_	Decay rate of *A*20_*mrna*_	0.0134	1/s
41.	*μ*_*ilc*_	Decay rate of *IL*10_*cyto*_	0.0073	1/s
42.	*μ*_*ile*_	Decay rate of *IL*10_*ext*_	8.736×10^−5^	1/s
43.	*μ*_*ilm*_	Decay rate of *IL*10_*mRNA*_	0.0234	1/s
44.	*μ*_*s*1*c*_	Decay rate of *SOCS*1_*cyto*_	3.591	1/s
45.	*μ*_*s*1*m*_	Decay rate of *SOCS*1_*mRNA*_	0.139	1/s
46.	*μ*_*s*3*c*_	Decay rate of *SOCS*3_*cyto*_	0.110	1/s
47.	*μ*_*s*3*m*_	Decay rate of *SOCS*3_*mRNA*_	0.0717	1/s
48.	*μ*_*tnc*_	Decay rate of *TNFα*_*cyto*_	0.009	1/s
49.	*μ*_*tnm*_	Decay rate of *TNFα*_*mrna*_	0.0126	1/s
50.	*p*	Transcription parameter	0.0364	*μ*M
51.	*s*_*m*_	Rate at which *NFκB* transcribes mRNA	0.237	1/s
52.	*SOCS*3_∞_	Relative effectiveness of *SOCS*3_*cyto*_ at inhibiting TNF*α* transcription	10.609	*μ*M
53.	*SOCS*_∞_	Relative effectiveness of *SOCS*1_*cyto*_ and *SOCS*3_*cyto*_ at inhibiting activation of STAT3	21.933	*μ*M
54.	*t*_*i*3_	Rate at which *IKK*/*IκBα*/*NF*-*κB* is broken down	4.669×10^−6^	1/s

#### TNF*α*

One of the main targets of gene expression of NF*κ*B is the pro-inflammatory cytokine TNF*α*. The first term of Eq (1) represents transcription of mRNA. There is evidence that Suppressor of Cytokine Signaling 3 (SOCS3), discussed in further detail below, plays a role in regulating the pro-inflammatory response by inhibiting TNF*α* mRNA and protein production, although the exact mechanisms by which this occurs is still unclear [19, 20]. We included a multiplier, not in the original equation by Maiti et al., in this first term to represent inhibition of mRNA production by SOCS3.

$$\begin{array}{cc} \frac{dTNF\alpha_{mrna}}{dt}= & \overset{\text{Transcription}\;\text{via}\;\text{NF}\kappa \text{B}}{\overset{︷}{\frac{s_{m}p\;NF\kappa B_{nuclear}}{c_{tf}+NF\kappa B_{nuclear}}}}\overset{\text{Inhibition}\;\text{by}\;\text{SOCS3}}{\overset{︷}{\left(\frac{1}{1+{\left(\frac{SOCS3_{cyto}}{SOCS3_{\infty }}\right)}^{2}}\right)}}-\overset{\text{Decay}}{\overset{︷}{\mu_{tnm}TNF\alpha_{mrna}}} \end{array}$$ (1)

#### JAK-STAT signaling

A hallmark of the anti-inflammatory response is the cytokine IL-10. Its gene is a target of NF*κ*B transcription and is involved in the regulation of the pro-inflammatory response. Some events related to IL-10 production and function are included in the model by Maiti et al. [6], but we expand the model to include a fuller view of the role of IL-10 and an important pathway it activates. [moved this paragraph one subsection down]

Aside from inhibitory functions, IL-10 signaling initiates the JAK-STAT signaling pathway, a primary mechanism through which the immune response mediates inflammation [21]. The protein tyrosine kinases JAK1 and Tyk2 are recruited to the IL-10/IL-10 receptor complex, shown in the third term of Eq (2). This creates a new complex, *IL*10/*R*/*JAK*1/*Tyk*2, Eq (5) [22]. The second term accounts for the possibility that the complex may break apart. JAK1 (Eq (3)) and Tyk2 (Eq (4)) concentrations are conserved, assuming enzyme-type dynamics. In light of the many components involved in creating this complex, we explored incorporating the various combinations of the binding steps, such as the individual receptor components, each of which bind to a specific tyrosine kinase. In the end, we decided to model the recruitment of JAK1 and Tyk2 to the IL-10/IL-10 receptor complex as one step; this still captures the appropriate dynamics without adding more parameters and equations. The last two terms of Eq (2) and all of Eqs (3) through (5) are our additions to the original model by Maiti et al., with terms representing activation of STAT3 through the Jak-STAT pathway adapted from Moya et al. [7].

$$\begin{array}{cc} \frac{dIL10/R}{dt} & =\overset{\text{IL-10}\;\text{binds}\;\text{to}\;\text{receptor}}{\overset{︷}{k_{ilrb}IL10_{ext}IL10R}}-\overset{\text{IL-10}\;\text{unbinds}\;\text{from}\;\text{receptor}}{\overset{︷}{k_{ilru}IL10/R}}\\ & -\overset{\text{Recruitment}\;\text{of}\;JAK1\;\text{and}\;Tyk2}{\overset{︷}{k_{iljb}IL10/R\;JAK1\;Tyk2}}+\overset{\text{Dissociation}\;\text{of}\;JAK1\;\text{and}\;Tyk2}{\overset{︷}{k_{ilju}IL10/R/JAK1/Tyk2}} \end{array}$$ (2)

$$\begin{array}{c} \frac{dJAK1}{dt}=-\overset{\text{Recruitment}\;\text{of}\;JAK1\;\text{and}\;Tyk2}{\overset{︷}{k_{iljb}IL10/R\;JAK1\;Tyk2}}+\overset{\text{Dissociation}\;\text{of}\;JAK1\;\text{and}\;Tyk2}{\overset{︷}{k_{ilju}IL10/R/JAK1/Tyk2}} \end{array}$$ (3)

$$\begin{array}{c} \frac{dTyk2}{dt}=-\overset{\text{Recruitment}\;\text{of}\;JAK1\;\text{and}\;Tyk2}{\overset{︷}{k_{iljb}IL10/R\;JAK1\;Tyk2}}+\overset{\text{Dissociation}\;\text{of}\;JAK1\;\text{and}\;Tyk2}{\overset{︷}{k_{ilju}IL10/R/JAK1/Tyk2}} \end{array}$$ (4)

$$\begin{array}{c} \frac{dIL10/R/JAK1/Tyk2}{dt}=\overset{\text{Recruitment}\;\text{of}\;JAK1\;\text{and}\;Tyk2}{\overset{︷}{k_{iljb}IL10/R\;JAK1\;Tyk2}}-\overset{\text{Dissociation}\;\text{of}\;JAK1\;\text{and}\;Tyk2\text{)}}{\overset{︷}{k_{ilju}IL10/R/JAK1/Tyk2}} \end{array}$$ (5)

#### STAT3

The IL-10/IL-10 receptor/JAK1/Tyk2 complex serves as a temporary docking station for inactive Signal Transducer and Activator of Transcription 3 (STAT3) [23]. Upon recruitment to the complex, STAT3 is activated and undergoes homodimerization, shown in the first term of Eq (6). Since activated STAT3 is a dimer formation, for every two inactive STAT3 molecules, one STAT3 complex is activated. This is accounted for by the factor of 2 in the first term of Eq (6). Maiti et al. modeled the recruitment and activation of STAT3 through binding of STAT3 to the IL-10/IL-10R complex without Jak1 and Tyk2. We included a multiplier representing inhibition by Suppressors of Cytokine Signaling 1 and 3 (SOCS1 and SOCS3), two IL-10 responsive genes as well as the second term of Eqs (8) and (9) which allow for the conservation of STAT3 in the model. SOCS1 inhibits JAK1 function by binding its SH2 domain to JAK1, preventing STAT3 from docking to the IL-10 complex. SOCS3 performs a similar role but docks to the receptor; since we do not model at the level of detail of specific binding locations, we model this inhibition as having the same result, which is preventing STAT3 from activating [24–26].

STAT3 translocates to the nucleus (second term of Eq (7)) and controls transcription of several IL-10 responsive genes. The main inhibitor of STAT3 function is PIAS3. The protein binds to activated STAT3, preventing further transcription [27]. We model this by including a deactivation term with rate *k*_*sni*_, shown in the second term of Eq (8). In the Maiti *et al.* model, activated STAT3 dimer returns directly to the cytosol. In our model, activated STAT3 first deactivates. Then assuming enyzme-type dynamics for all states of STAT3, the transcription factor is conserved, and deactivated nuclear STAT3 returns to the cytosol in the last term of Eq (9).

$$\begin{array}{cc} \frac{dSTAT3_{i}}{dt} & =-\overset{\text{STAT3}\;\text{activation}}{\overset{︷}{2k_{stat}IL10/R/JAK1/Tyk2\;STAT3_{i}^{2}}}\overset{\text{Inhibition}\;\text{by}\;\text{SOCS1/3}}{\overset{︷}{\left(\frac{1}{1+{\left(\frac{SOCS1_{cyto}+SOCS3_{cyto}}{SOCS_{\infty }}\right)}^{2}}\right)}}\\ & +\overset{\text{Moves}\;\text{to}\;\text{cytosol}}{\overset{︷}{k_{snicyto}STAT3_{ni}}} \end{array}$$ (6)

$$\begin{array}{cc} \frac{dSTAT3_{a}}{dt} & =\overset{\text{STAT3}\;\text{activation}}{\overset{︷}{k_{stat}IL10/R/JAK1/Tyk2\;STAT3_{i}^{2}}}\overset{\text{Inhibition}\;\text{by}\;\text{SOCS1/3}}{\overset{︷}{\left(\frac{1}{1+{\left(\frac{SOCS1_{cyto}+SOCS3_{cyto}}{SOCS_{\infty }}\right)}^{2}}\right)}}\\ & -\overset{\text{Moves}\;\text{to}\;\text{nucleus}}{\overset{︷}{k_{sa}STAT3_{a}}} \end{array}$$ (7)

$$\begin{array}{c} \frac{dSTAT3_{n}}{dt}=\overset{\text{Moves}\;\text{to}\;\text{nucleus}}{\overset{︷}{k_{sa}STAT3_{a}}}-\overset{\text{Deactivation}}{\overset{︷}{k_{sni}STAT3_{n}}} \end{array}$$ (8)

$$\begin{array}{c} \frac{dSTAT3_{ni}}{dt}=\overset{\text{Deactivation}}{\overset{︷}{k_{sni}STAT3_{n}}}-\overset{\text{Moves}\;\text{to}\;\text{cytosol}}{\overset{︷}{k_{snicyto}STAT3_{ni}}} \end{array}$$ (9)

#### SOCS

The inclusion of SOCS, represented in Eqs (10) through (13), is also novel to our model as compared to that by Maiti et al. Suppressors of Cytokine Signaling 1 and 3 (SOCS1, SOCS3) are upregulated via STAT3 transcription and translation, first two terms of Eqs (10) and (11), respectively [18, 28]. The last terms of these two equations represent natural degradation of the mRNA.

$$\begin{array}{c} \frac{dSOCS1_{mRNA}}{dt}=\overset{\text{Gene}\;\text{transcription}}{\overset{︷}{k_{s1st}STAT3_{n}}}-\overset{\text{Translation}}{\overset{︷}{k_{s1}SOCS1_{mRNA}}}-\overset{\text{Decay}}{\overset{︷}{\mu_{s1m}SOCS1_{mRNA}}} \end{array}$$ (10)

$$\begin{array}{c} \frac{dSOCS3_{mRNA}}{dt}=\overset{\text{Gene}\;\text{transcription}}{\overset{︷}{k_{s3st}STAT3_{n}}}-\overset{\text{Translation}}{\overset{︷}{k_{s3}SOCS3_{mRNA}}}-\overset{\text{Decay}}{\overset{︷}{\mu_{s3m}SOCS3_{mRNA}}} \end{array}$$ (11)

$$\begin{array}{c} \frac{dSOCS1_{cyto}}{dt}=\overset{\text{Translation}}{\overset{︷}{k_{s1}SOCS1_{mRNA}}}-\overset{\text{Decay}}{\overset{︷}{\mu_{s1c}SOCS1_{cyto}}} \end{array}$$ (12)

$$\begin{array}{c} \frac{dSOCS3_{cyto}}{dt}=\overset{\text{Translation}}{\overset{︷}{k_{s3}SOCS3_{mRNA}}}-\overset{\text{Decay}}{\overset{︷}{\mu_{s3c}SOCS3_{cyto}}} \end{array}$$ (13)

We used this model that includes both pro- and anti-inflammatory signaling pathways to provide a fuller picture of the spectrum of activation that can occur within a macrophage. In the following pages, we discuss how this model was implemented and compared to the ABM.

### Parameters & initial conditions for ODE model

With the parameter values from the original Maiti et al. model [6] and some hand-tuning due to the added model components, we are able to reproduce dynamics similar to those presented in the Maiti et al. paper with an LPS initiated response for the TNF*α* variable and early time points for IL-10 variable. We used the initial conditions and parameter values from Maiti et al. as a starting point for our ODE model and their data as a frame of reference. However, since the data provided by the authors was processed data and did not align exactly with our model variables we did not fit to this data. Since the model simulations by Maiti et al. were initialized with LPS, once the hand-tuned parameter set was obtained, the model was run for 1,000 hours with no LPS. The ending values of these simulations for each variable were determined to be the baseline initial conditions, representing a state of no macrophage activation. Initial conditions are provided in the supplementary code.

With our model we focused on inflammation initialized with TNF*α*, using the same scenario for the initial conditions for both the agent-based and ODE models (calibrating experiment) we determined parameters that gave plausible dynamics that were similar for both model. This was our initial guess for additional optimization of the ODE model to obtain good initial agreement between the ABM and ODE model behavior. To determine the final parameter set we used the ABM output of M1 and M2 activation, PIM and AIM from the calibrating experiment as “data” and fit the corresponding ODE model variables to these outputs. Parameters were allowed to vary a factor of 2 above and below their starting values. We tested other ranges allowing them to vary up to 100 times above and below, but did not see an significant difference in the results. The details of this process are provided in the “Calibrating experiment and scenarios” section. Final parameter values are given in Table 1.

### Modeling multiple macrophages

**Fig 2:**
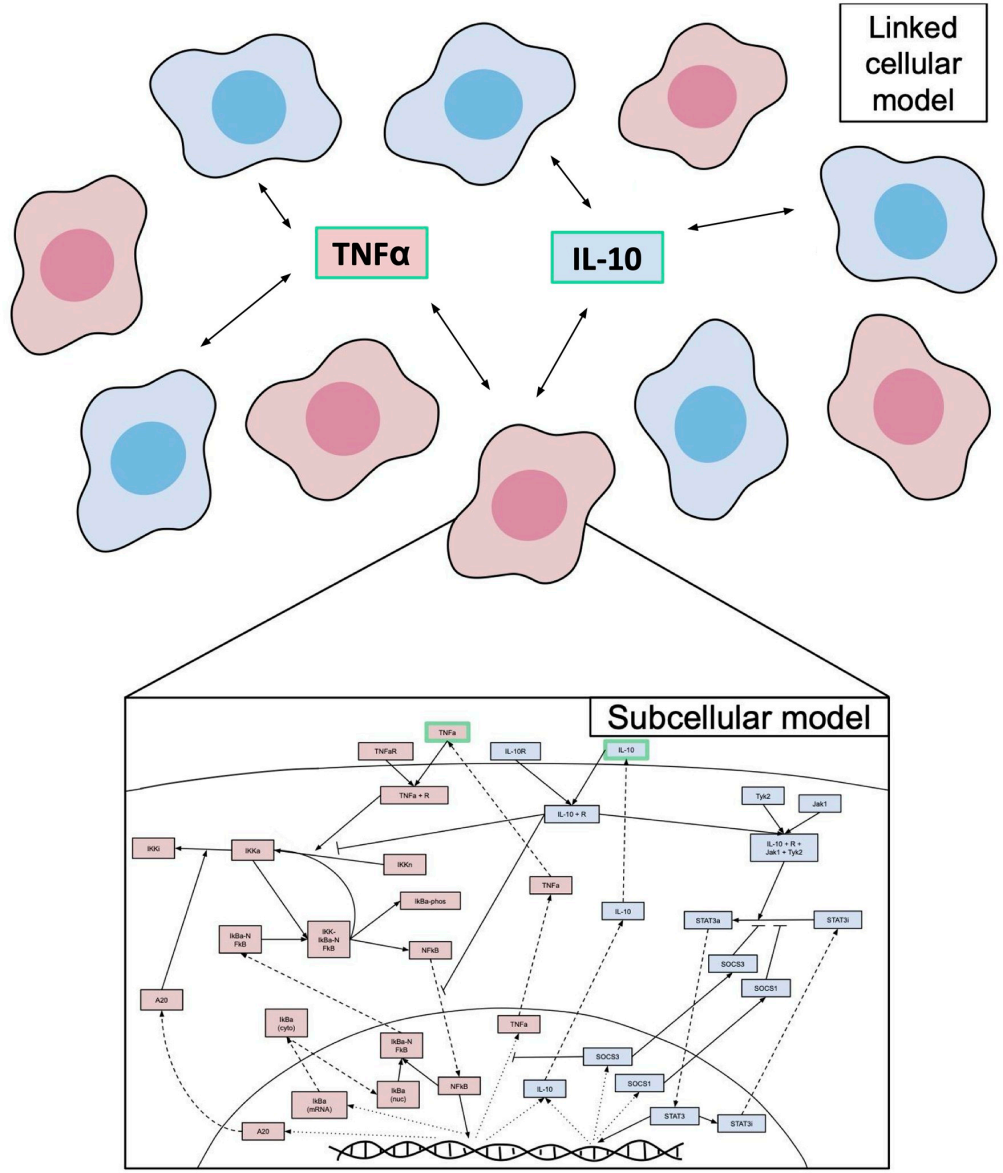
Cellular and subcellular scales in the model are linked by extracellular signals. A representation of the multiple macrophages ODE model, in which each macrophage is a compartment with its own set of subcellular signaling pathways, and all ten macrophages share external stimuli TNF*α*, and IL-10.

The equations described in the section above represent the pathways in a single macrophage. To model recruitment and cell lifespan, we extended the model to represent ten macrophages. These macrophages share the same extracellular components: IL-10 and TNF*α*. Fig 2 shows a visualization of this compartmental model. Each macrophage is represented by the same parameter values and is randomly assigned a lifespan. At the end of each cell’s lifespan, the variables in the signaling pathway are returned to a naive state to represent the recruitment of a naive cell. Initial conditions are determined by the simulations described in the following sections.

Our aim in constructing a model of multiple macrophages was to examine how macrophages in close proximity behave in response to extracellular stimuli while still utilizing the ODE structure. Resulting dynamics of variables that exist in each model can be viewed separately or averaged together to obtain the average behavior across all macrophages. Furthermore, we chose the number of macrophages to be an arbitrarily small number to represent a localized response to pro- and anti-inflammatory signaling. We wanted the number of macrophages to be small enough to reduce simulation time. We also simulated the model with 50 macrophages and the differences were not significant, so we presented the results for 30 macrophages and did continue to simulate larger populations. Also, given that there is stochasticity in the model, we ran the model 30 times and plotted the average results in our figures.

### Agent-based M1/M2 model

Our ABM tracks pro- and anti-inflammatory mediators (PIM and AIM, respectively), M0, M1, and M2 macrophages, and SOCS on a 40-by-40 grid, implemented using object-oriented programming in MATLAB (code provided in the supplement). We adjusted the scale of the grid by factors of 1/2 and 10 and the results did not significantly change, so we continued with a grid size of 40-by-40. Macrophages are mobile agents with M1/M2 activation and SOCS levels as associated attributes. Each macrophage may take up one patch, and pro- and anti-inflammatory mediators are measured by amount on each patch, diffusing across the grid over time. We do not specifically model particular cytokines, but rather the general levels of pro- and anti-inflammatory mediators. The model can be initialized with varying levels of any of these components and simulated to obtain the resulting dynamics.

**Fig 3:**
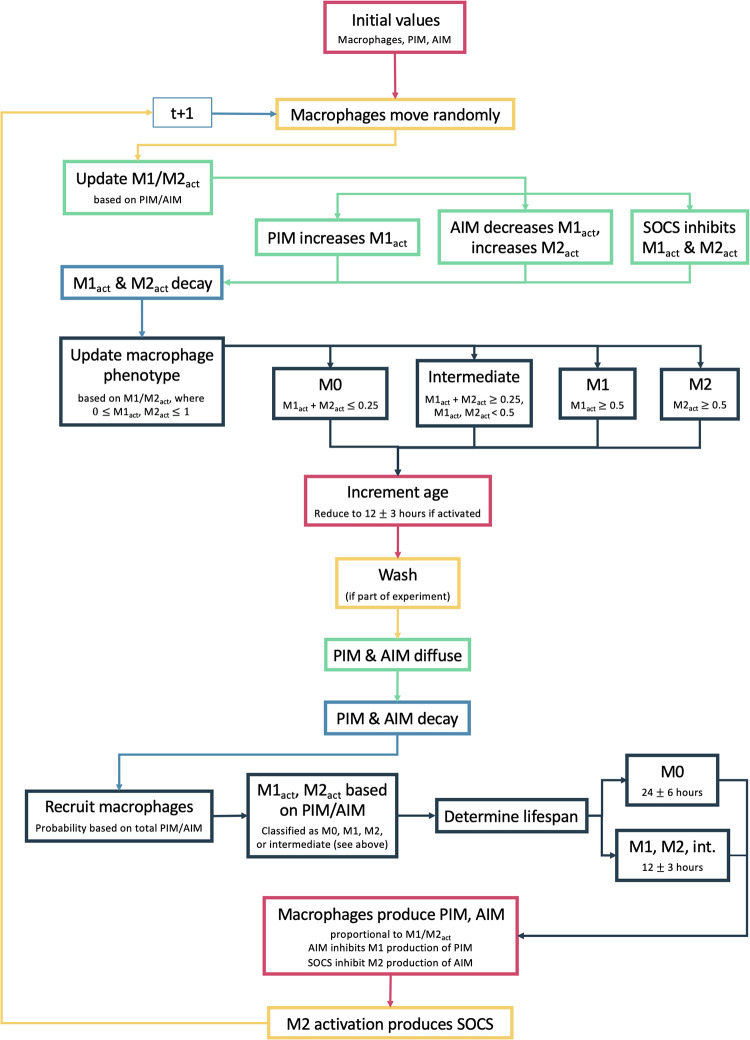
Flow chart for ABM rules. Description of steps in ABM for each iteration of the simulation.

Fig 3 summarizes the steps taken during every iteration of the simulation. Each iteration represents 20 minutes; With this iteration time and the parameter values chosen, we are able to achieve appropriate timescales for macrophage activation. These steps are based on the same interactions described in the ODE model. First, cells that have reached the end of its assigned lifespan are removed from the model. Remaining macrophages will randomly select one of its eight surrounding patches to move to. If the patch already contains a macrophage, it does not move. M1 and M2 activation of each macrophage, which sum to 1, is updated based on the amount of PIM and AIM on the patch containing the macrophage as well as the level of SOCS associated with the macrophage. PIM increases M1 activation and AIM increases M2 and decreases M1 activation through Hill-type functions. SOCS inhibits M1 and M2 activation through a multiplier in these functions. If M1+M2 is above 1 in this step, both are re-scaled to achieve the same proportions but sum to 1. M1 and M2 activation also have first-order decay rates.

Based on M1 and M2 activation levels, the macrophage phenotype is then determined. If M1 and M2 activation sum to 0.25 or less, the phenotype is M0. If either M1 activation or M2 activation is greater than or equal to 0.5, the macrophage has an M1 or M2 phenotype, respectively. Otherwise, the phenotype is considered intermediate. In our simulations, we focus on M1 and M2 activation levels rather than the phenotype since they are continuous variables on a scale from 0 to 1 such that 0 ≤ M1 + M2 ≤ 1, but phenotype can be helpful for visualization and classification.

In the next step, the age of the macrophage is increased. If the macrophage is newly classified as activated (M1, M2 phenotype), the lifespan is increased, since activated macrophages have longer lifespans. PIM and AIM diffuse such that for every amount in a patch, the amount is distributed evenly between that patch and the eight surrounding patches. PIM, AIM, and SOCS then decay at a first-order rate.

New macrophages are the introduced into the grid through “recruitment” based on the amount of PIM and AIM. The probability of recruitment is calculated by a Hill-type function where PIM has a greater influence than AIM. The model generates a grid of randomly-generated number between 0 and 1 for each patch; if the generated number is less than the recruitment probability calculated for that patch, a macrophage is placed there. M1 and M2 activation levels, phenotype, and age for each recruited macrophage are determined by the PIM and AIM levels on the patch.

Finally, macrophages produce PIM, AIM, and SOCS. PIM is produced proportionally to the M1 activation level, inhibited by AIM through a nonlinear multiplier. AIM is produced proportionally to M1 activation and to M2 activation, inhibited by SOCS through a nonlinear multiplier. SOCS is produced proportionally to M2 activation. A small amount of noise is introduced into the production rate to account for variability between macrophages. The time step is complete and the process starts over with a new iteration.

### Calibrating experiment and scenarios

To compare the models to each other, we implemented the same scenario, which we called a “calibrating experiment,” in each model and tuned the ABM and ODE model results so that PIM & AIM and M1 & M2 activation results were similar to their corresponding components (see section describing ODE model parameters for process). These ODE model components were extracellular TNF*α* & IL-10 and TNF*α* mRNA & IL-10 mRNA, respectively. We chose M1 and M2 activation to be represented by TNF*α* and IL-10 mRNA, respectively, since mRNA is produced via downstream signaling initiated by the surrounding environment and also results in specific proteins that are secreted from the cell. Thus, mRNA associated with the cell’s phenotype both reflects and drives macrophage polarization.

Tuning parameters so that the ABM and ODE model returned similar dynamics in the calibrating experiment allowed us to obtain similar behavior at baseline and compare the results of more complicated experiments. We chose this scenario to be a single macrophage with a high pro-inflammatory stimulus and without cell death. In the ODEs, initial conditions were established such that all variables are at baseline levels, to represent an M0 macrophage. TNF*α*, the variable representing a pro-inflammatory stimulus in the ODE model, was set to 10 pg/mL, consistent with experimental methods [29].

For the ABM calibrating experiment only, we used a 3-by-3 grid with one naive macrophage so that the cell could move but interact only with the mediators in its immediate proximity. A naive macrophage in this model is defined as having activation M1 + M2 < 0.25; M1 and M2 activation were randomly chosen with bounds that satisfy this condition. Pro-inflammatory mediators do not have specific units but after exploratory simulations, we considered an amount of 30 in the center space of the grid to be sufficient to mount an inflammatory response.

**Fig 4:**
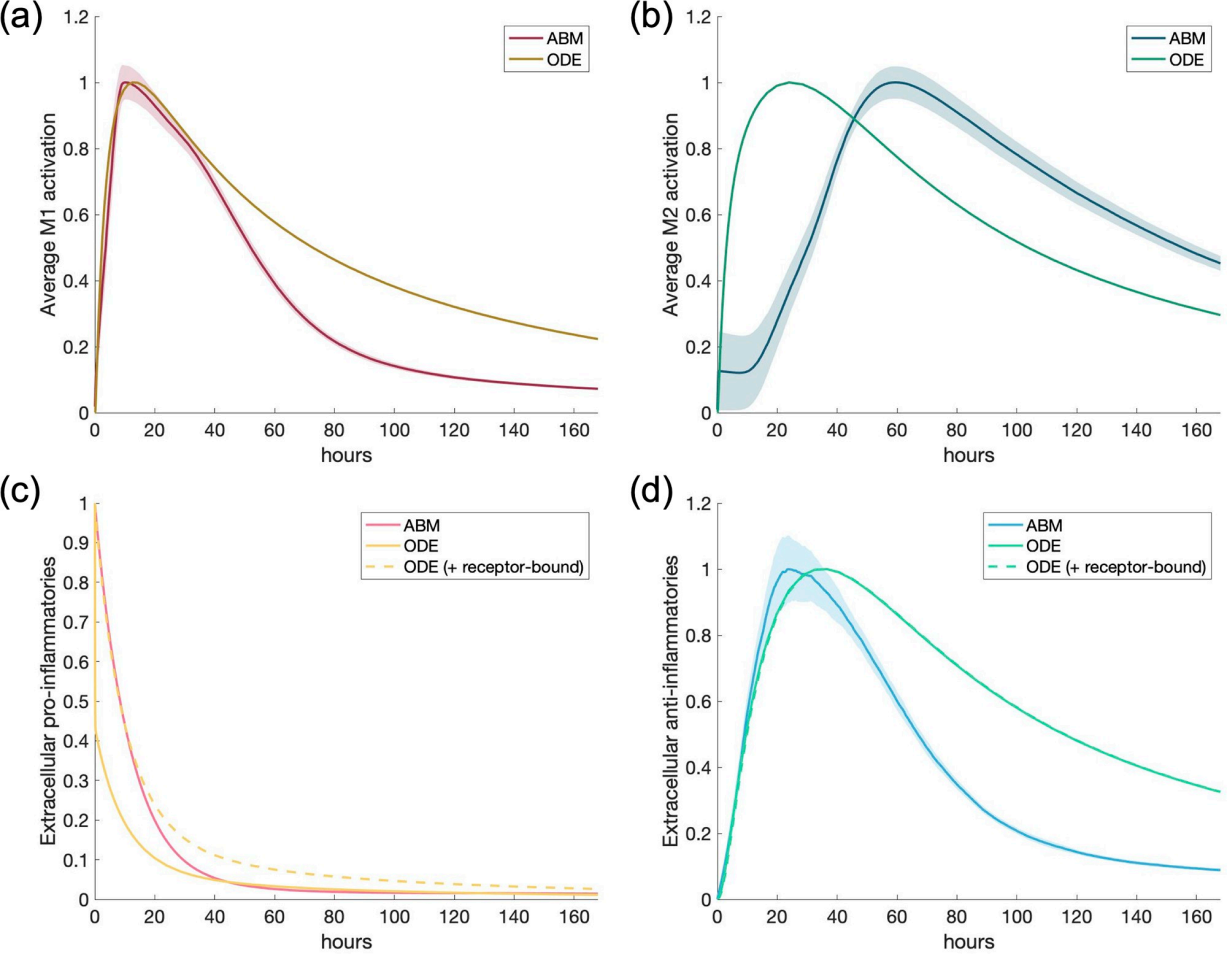
Results for calibrating experiment show good agreement between models. Calibrating experiment: single macrophage activated by a pro-inflammatory stimulus. ABM and ODE results are shown on the same plots for comparison. All transients are scaled by their maximums. Dotted lines represent extracellular TNF*α* or IL-10 with receptor-bound TNF*α* or IL-10, respectively. (a) M1 activation, (b) M2 activation, (c) pro-inflammatory mediators, (d) anti-inflammatory mediators.

**Fig 5:**
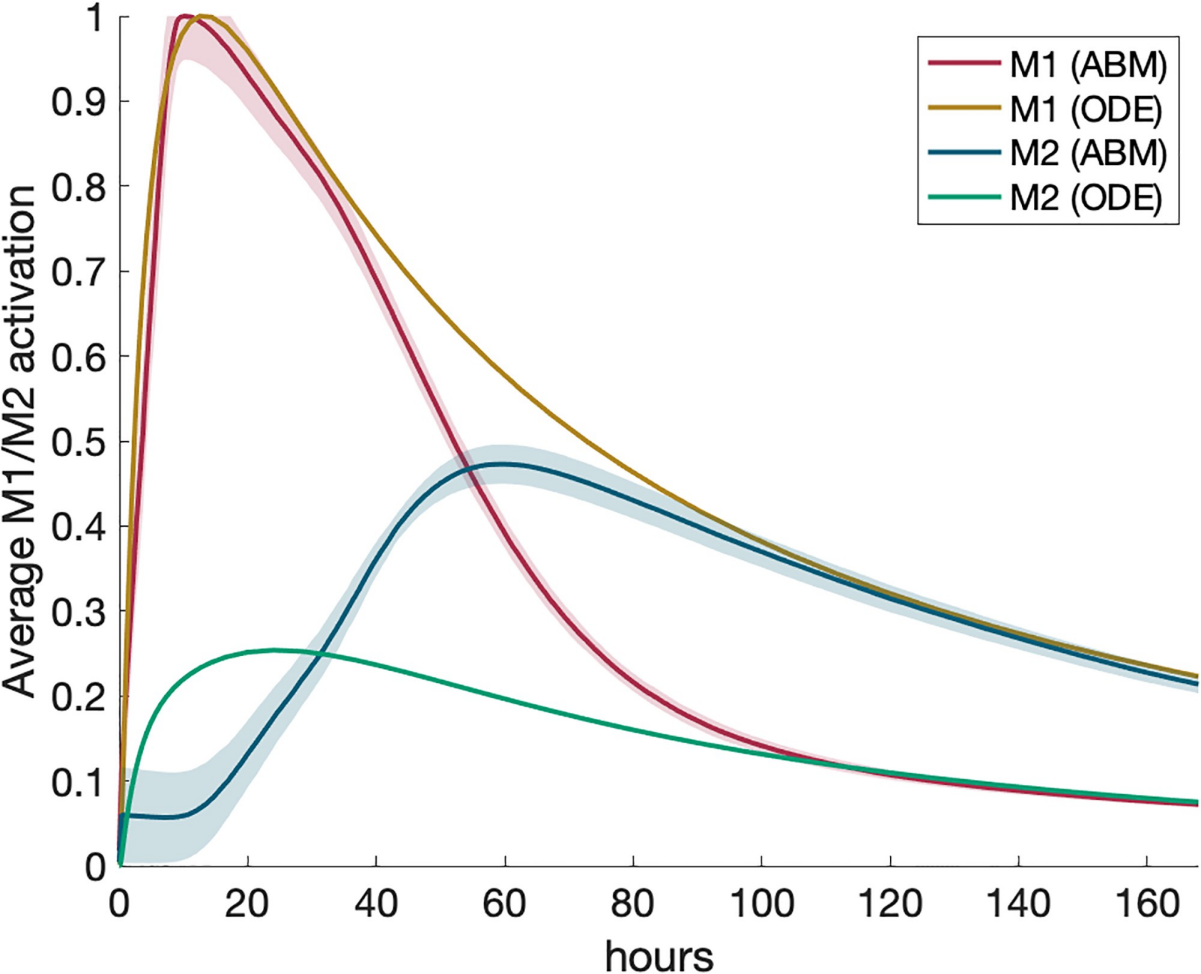
Results for M1 activation relative to M2 activation show good agreement between models. Calibrating experiment: M1 and M2 activation resulting from the calibrating experiment. ODE results are scaled by the maximum M1 activation to compare to activation in the ABM, which is bound by 0 and 1.

In Figs 4 and 5 and in our results after the fit, we show two outputs from the ODE model: when only extracellular TNF*α* and IL-10 are considered, and when both extracellular and receptor-bound TNF*α* and IL-10 are considered, since this may not be a negligible amount. Observations from the differences between the two simulations are considered in the discussion section.

In the calibrating experiment, we set the ODE and ABM parameters and initial conditions such that a single macrophage would exhibit similar M1 and M2 behavior when initialized with PIM (process described in Methods section). Fig 4 shows the results of this simulation. All transients are normalized for comparison because the units in the models vary. To do this, we scaled each transient by its maximum. The results of the ABM in Fig 4 is the result of 30 simulations; on the other hand, the ODE model with a single macrophage is deterministic and thus only one simulation is necessary.

Note that activation of the M1 response and total pro-inflammatories (bound and unbound) are similar throughout the simulation. Anti-inflammatories are inline during the initial phase of the response. However, M2 activation occurs earlier in the ODE than in the ABM, but given the difference in the level of detail for this phase of the inflammatory response this was not surprising and this difference was taken into consideration in latter results. Adding receptor-bound TNF*α* and IL-10 to their extracellular counterparts did not make a significant difference in the results. We also considered the magnitudes of M1 and M2 activation in relation to each other, shown in Fig 5. M1 and M2 activation in the ABM are, by definition, bound between 0 and 1. To compare with the ABM, we scaled TNF*α* and IL-10 mRNA in the ODE by the maximum of TNF*α*. Peak M2 activation in both the ABM and ODE are about one-quarter to half the peak M1 activation. This shows important dynamics observed in both models.

Once the parameters were set and the calibrating experiment was simulated, we changed the initial conditions to represent six additional scenarios, which will be described in greater detail below. Parameter values are kept constant throughout all simulations to signify that all macrophages have the same capabilities; only initial conditions are adjusted for each scenario to represent its current activation state. First, we used the same single-macrophage model as described above but with an anti-inflammatory stimulus (Scenario 1). Then, using the 40-by-40 grid for the ABM and ten-macrophage model for the ODE, we incorporated recruitment/turnover and cell lifespan. For these larger models, we simulated the following scenarios, the results of which will be discussed in the following section:

2. Naive macrophages with large pro-inflammatory stimulus
3. Naive macrophages with large anti-inflammatory stimulus
4. M1 macrophages with anti-inflammatory stimulus
5. Half M1, half M2 macrophages
6. Pro-inflammatory stimulus, wash at hour 12, then anti-inflammatory stimulus

For the ODE model, a pro- or anti-inflammatory stimulus is considered to be 10 pg/mL, as in the calibrating experiment, unless otherwise stated. For the ABM, the corresponding value is 30. Amounts in the ABM are unitless; as previously stated, exploratory simulations showed that this was sufficient to initiate an immune response. SOCS is always initialized at zero. Code to run the calibrating experiment for the ODE model and ABM is in the S1 File.

### Sensitivity analysis

We performed a one-at-a-time sensitivity analysis (SA) on both the ABM and ODE models to identify the key parameters and initial conditions that drive macrophage activation and expression of pro- and anti-inflammatory mediators. Using the single-macrophage calibrating experiment, we perturbed each parameter (except for those related to lifespan) and initial condition for both models by 10% above or below the original value and calculated the percent change for key variables and multiple time points. Parameters and their descriptions are shown in Tables 1 and 2 for the ODE model and ABM, respectively. For the ABM, AIM and SOCS start out at zero for the calibrating experiment, and M1 and M2 activation levels are randomly defined within the range of an M0 macrophage. Thus we vary PIM and M0 for the calibrating experiment, and vary AIM in Scenario 1 to obtain sensitivity analysis results for AIM.

**Table 2:** ABM final parameter values and descriptions.

Parameter	Value	Description
ImmuneProInflammatoryRate	0.35	Rate at which M1s produce PIM
ImmuneAntiInflammatoryRate	0.85	Rate at which M2s produce AIM
ImmuneM1AntiInflammatoryRate	0.175	Rate at which M1s produce AIM
ProInflammatoryDecayRate	0.03	Rate at which PIM decay
AntiInflammatoryDecayRate	0.03	Rate at which AIM decay
SOCSDecayRate	0.03	Rate at which SOCS decay
PIMNegativeFeedbackRate	0.002	Rate at which M1 activation decays
AIMNegativeFeedbackRate	0.003	Rate at which M2 activation decays
RecruitmentMMTerm	30	Regulates effectiveness of macrophage recruitment by PIM & AIM
AIMRecruitScale	0.1	Rate at which AIM recruit macrophages
PIMActivationScale	0.75	Regulates effectiveness of M1 activation of newly recruited cells by PIM
AIMActivationScale	0.75	Regulates effectiveness of M2 activation of newly recruited cells by AIM
AIMInfinity	1	Regulates effectiveness of AIM in inhibiting M1 activation of newly recruited cells by PIM
M1ActivationRate	0.05	Rate at which M1 activation is increased by PIM
M1ActHillParameter	1	Regulates effectiveness of increasing M1 activation via PIM
M2ActScalar	0.065	Rate at which M2 activation is increased by AIM
M2ActHillParameter	0.85	Regulates effectiveness of increasing M2 activation via AIM
M1AIMInfinity	0.05	Regulates effectiveness of AIM in inhibiting M1 activation of local cells by PIM
M1DecreaseViaAIM	0.01	Rate at which M1 activation is decreased by AIM
M1DecreaseViaAIMHill	0.4	Regulates effectiveness of decreasing M1 activation by AIM
SOCSProductionRate	4	Rate at which AIM produce SOCS
AIMSOCSHill	5	Regulates effectiveness of SOCS production by AIM
M1SOCSInfinity	4	Regulates effectiveness of inhibiting M1 activation by SOCS
M2SOCSInfinity	7	Regulates effectiveness of inhibiting M2 activation by SOCS
AIMSOCSInfinity	0.01	Regulates effectiveness of inhibiting M2 production of AIM production by SOCS

In this work, we are most interested in M1/M2 activation and pro- and anti-inflammatory mediators; thus, we track results for the corresponding model variables. Since lifespan is not considered, TNF*α*-induced dynamics can persist for the four variables for about 1000 hours. Thus, we obtained SA results for the following time points: 50, 100, 250, 500, 750, and 1000 hours.

## Results

We simulated equivalent scenarios in an ODE model and an agent-based model of M1/M2 activation in response to general inflammatory stimuli. In this section we compare the results of the two models to shed light on the benefits of each model type and, in particular, examine whether the incorporation of a spatial component through an ABM or the incorporation of hallmark signaling pathways through an ODE improve the value of the models in understanding immune system dynamics. All results shown are the average of 30 simulations except the single-macrophage simulations, which is deterministic since age is not a factor.

### Scenario 1: Macrophage with anti-inflammatory stimulus

**Fig 6:**
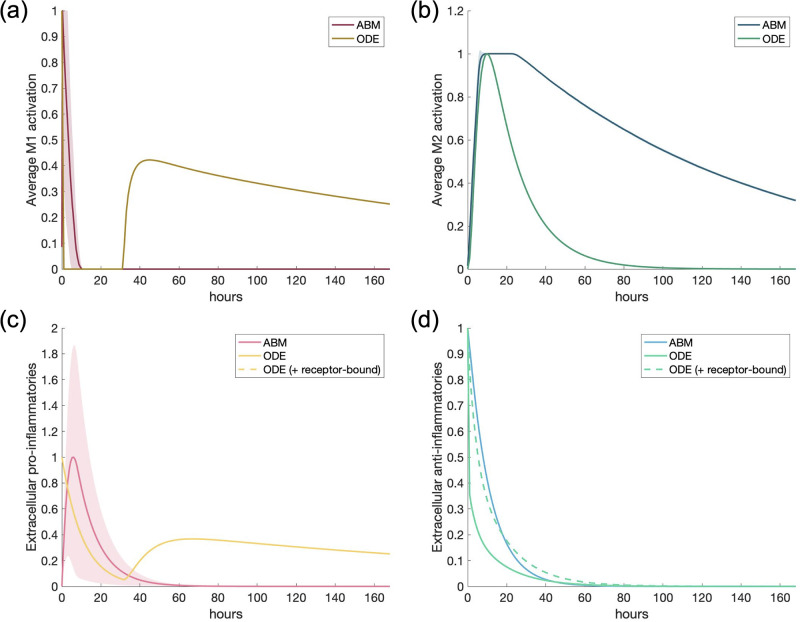
Scenario 1: Simulation of single response to an anti-inflammatory stimulus. All transients are scaled individually by their maximums. (a) M1 activation, (b) M2 activation, (c) PIM, (d) AIM.

For the first scenario, we used the same structure of a single macrophage as in the calibrating experiment. Instead of a pro-inflammatory stimulus, we use the same amount as in the calibrating experiment but as an anti-inflammatory stimulus. Fig 6 shows the results of this simulation. AIM and M2 activation behave roughly the same; in the ABM, M2 activation decreases slower than in the ODE, which is consistent with the differences in these models shown in the calibrating experiment.

Including receptor-bound mediators in the ODE reveals a slower decrease in AIM over time that matches the ABM. A small increase in M1 activation and PIM occurs later in time in the ODE model; this is due to trace amounts of NF*κ*B in the baseline levels of the cell that result in a small amount of TNF*α* production downstream. Additionally, we noted that simulating a single macrophage in the ABM shows consistent results for each of the 30 simulations, since the shaded regions around the curves, representing standard deviation, are very small or nearly zero, except for PIM. The increased standard deviation for PIM is likely due to the fact that there is not much PIM in the simulation in the first place, due to the AIM activating macrophages more towards an M2 phenotype.

**Fig 7:**
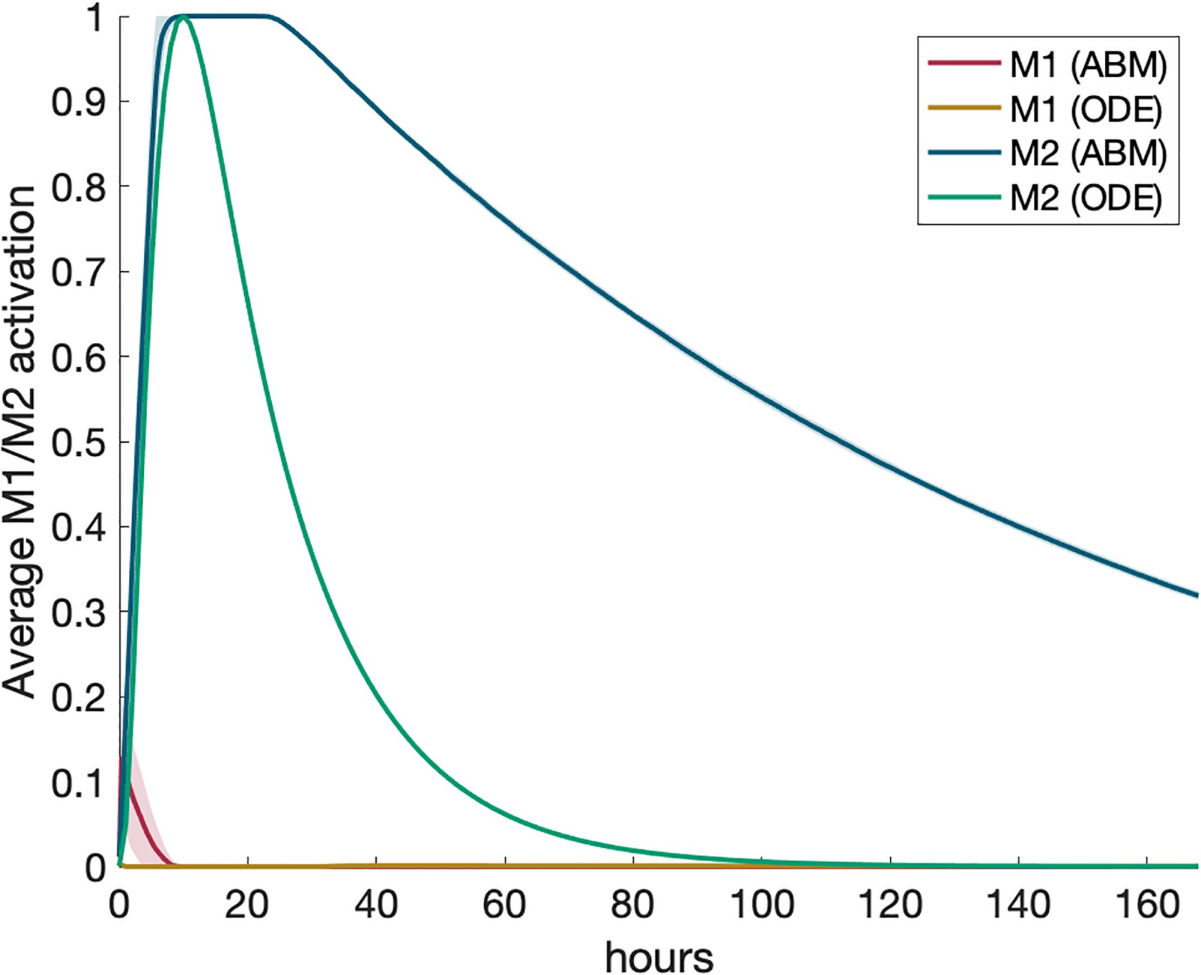
For anti-inflammatory stimulus, M1 and M2 are comparable between models. Scenario 1: M1 and M2 activation response to an anti-inflammatory stimulus. ODE and ABM results are scaled by the maximum M2 activation to compare to maximum M1 activation, which is nearly nonexistent in comparison to M2.

Fig 6(c) shows that some PIM is produced in the ABM due to a small percentage of M1 activation existing in the naive macrophages (see Fig 3 to see how naive macrophages are defined), but both models show a decrease to zero in the presence of a large concentration of AIM. Similarly to the calibrating experiment in Fig 5, we show M1 activation in relation to M2 activation in Fig 7 to better visualize the magnitude of the pro-inflammatory response, which is very small in relation to the much larger anti-inflammatory stimulus.

### Scenario 2: Multiple macrophages with pro-inflammatory stimulus

**Fig 8:**
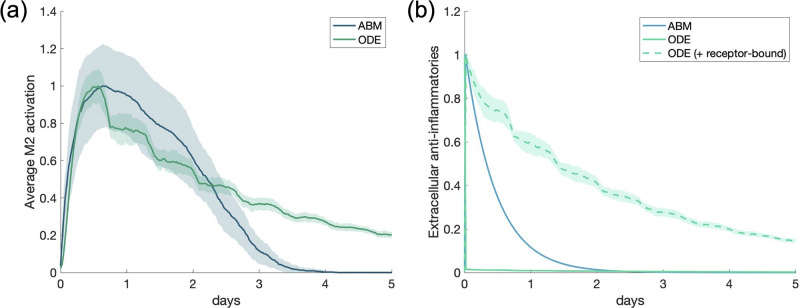
Multi-cell response to a pro-inflammatory stimulus. Scenario 2: M1/M2 response to model of multiple macrophages, activated by an initial amount of pro-inflammatory mediators. All transients are scaled individually by their maximums. “ODE (+ receptor-bound)” is the sum of the extracellular cytokine plus the receptor-bound cytokine. (a) M1 activation, (b) M2 activation, (c) PIM, (d) AIM.

We then introduced recruitment/turnover and cell lifespan to the simulations. In the ABM, the grid was expanded to 40-by-40 with ten M0 macrophages initially, and the recruitment feature was turned on. Naive and activated macrophages were randomly assigned lifespans of 24 ± 6 and 48 ± 12 hours, respectively [30, 31]. In the ODE, all macrophage compartments were utilized. To ensure that differences between model types are not driven by lifespan, the lifespan defined in the ODE model was calibrated such that the ABM and ODE M1 timescales in response to pro-inflammatory stimuli in Scenario 2 and M2 timescales in response to anti-inflammatory stimuli in Scenario 3 are roughly the same between the ODE model and ABM. The resulting calibrated lifespan was 16 ± 2 hours. In this scenario, we introduced a large pro-inflammatory stimulus of 30 into the model. Results are shown in Fig 8.

In this scenario, M1 activation and PIM exhibit similar dynamics while M2 activation and AIM differ in timescale between model types. M1 and M2 activation peak slightly earlier in the ODE than ABM and M2 and AIM persist longer in the ODE model than in the ABM. This persistence of one model over the other is not reflected in the calibrating experiment; thus, the persistence could be due to dynamics unique to the larger number of macrophages in the ODE model that are not equivalent in the ABM. Including receptor-bound TNF*α* (Fig 8(c) and 8(d)) impacts PIM but not AIM. Our ODE model shows that when naive macrophages are introduced into an environment with a high concentration of TNF*α*, receptors quickly bind to free TNF*α*. Therefore, unbound extracellular TNF*α* in the ODE did not compare as well to PIM in the ABM, since receptors are not modeled in the ABM. When receptor-bound TNF*α* was added to the PIM total shown in Fig 8, the dynamics matched up much more closely to the ABM. Fig 8(d) shows almost no difference between extracellular IL-10 only and extracellular IL-10 with receptor-bound IL-10, suggesting that accounting for both populations matters more when a large amount of extracellular mediators is introduced rather than the resulting dynamics are observed over time. Standard deviations, shown as the shaded regions in the figures, are narrow and similar to the single-macrophage simulations, showing that our choices for the number of macrophages and total simulations for both models are likely capturing the overall behavior of the model.

### Scenario 3: Multiple macrophages with anti-inflammatory stimulus

**Fig 9:**
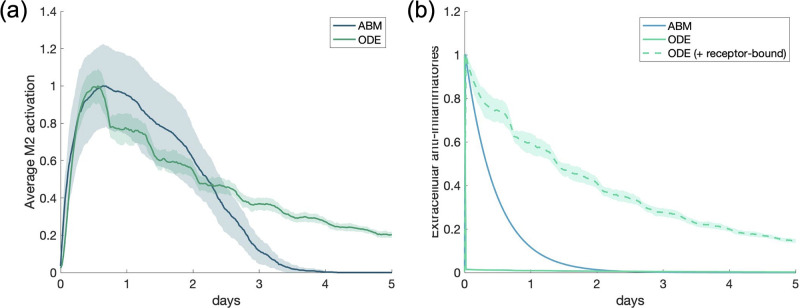
Multi-cell response to anti-inflammatory mediators. Scenario 3: M1/M2 response to M1 macrophages activated by an initial amount of anti-inflammatory mediators. All transients are scaled individually by their maximums. “ODE (+ receptor-bound)” is the sum of the extracellular cytokine plus the receptor-bound cytokine. (a) M2 activation, (b) AIM.

The same initial conditions were used for this scenario as in the previous one, except instead of a pro-inflammatory stimulus, an equivalent anti-inflammatory stimulus was introduced into the system. Results are shown in Fig 9. PIM and M1 activation were very small compared to AIM and M2 activation, so we do not show their dynamics.

M2 activation in the two models are relatively similar, with the ODE showing a slightly longer tail after the peak of activation for both M2 activation and AIM. This is paired with a slower decrease of IL-10 (AIM) when receptor-bound IL-10 is taken into account, similarly to PIM in the previous scenario. For a large anti-inflammatory stimulus, more similar dynamics are observed between both models when the ODE transient includes receptor-bound IL-10.

### Scenario 4: M1 macrophages with anti-inflammatory stimulus

Next we examined what would happen to an M1 environment when an anti-inflammatory stimulus is introduced into the system. We first needed to determine what this M1 environment would look like as initial conditions that could be used to begin the simulation.

For the ODE model, we set all ten macrophages to an M1 phenotype based on the maximum activation that occurs in the calibrating experiment. This maximum occurs around hour 32, so we used the variable values at this time as the initial conditions for all macrophages. We then set extracellular TNF*α* to zero and added a high concentration of IL-10 (the same amount as in Scenario 3) and ran the simulation.

**Fig 10:**
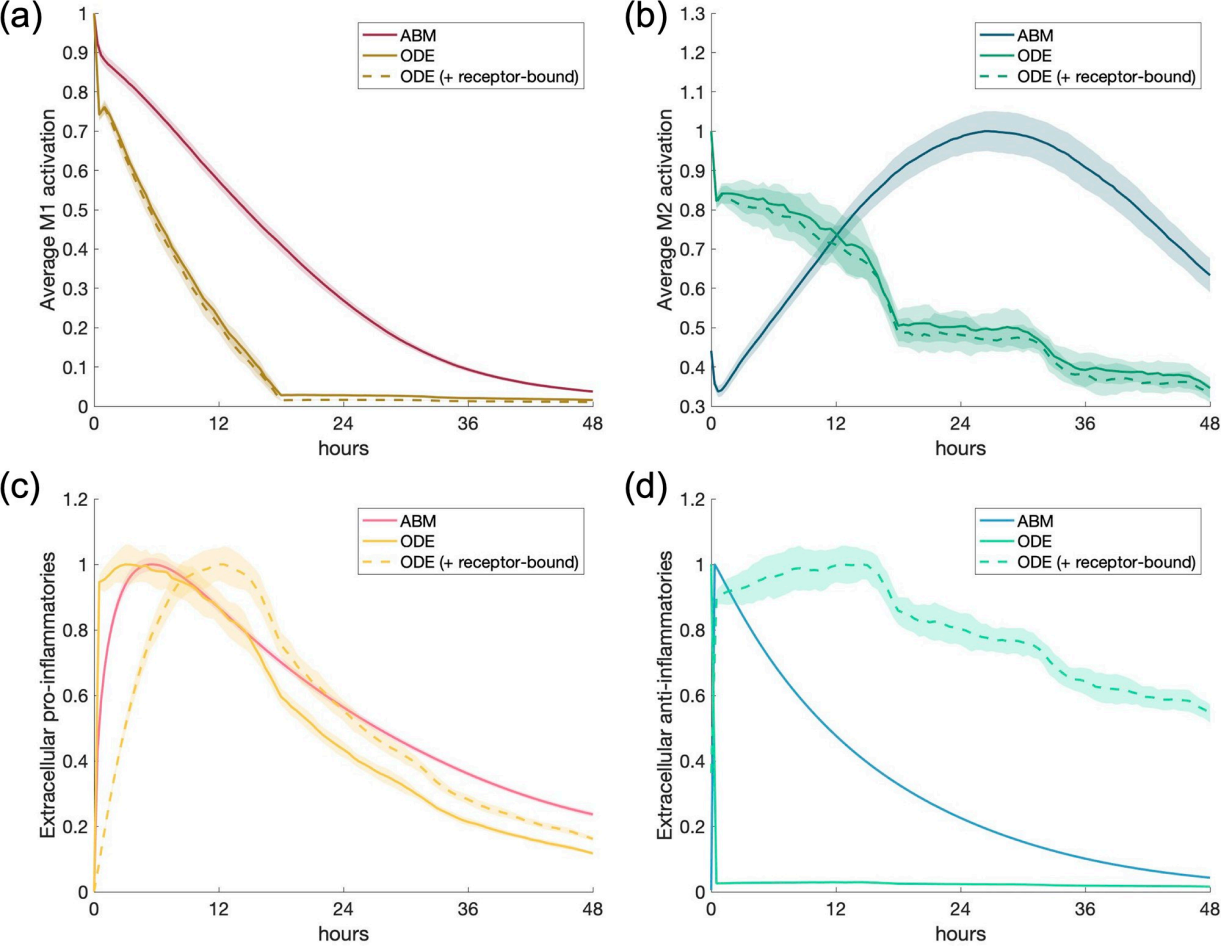
Multi-cell response to AIM in M1 environment. Scenario 4: M1/M2 response to anti-inflammatory stimulus introduced into an M1-polarized system. Transients are scaled individually by their maximums. “ODE (+ receptor-bound)” is the sum of the extracellular cytokine plus the receptor-bound cytokine. (a) M1 activation, (b) M2 activation, (c) PIM, (d) AIM.

For the ABM, M1 macrophages are defined as having M1_act_ > 0.5 and produce pro- and anti-inflammatory mediators proportional to their activation. To account for recruitment, the equivalent of which in the ODE model is turnover to naive initial conditions, we introduced into the system the number of M1 macrophages at the time when M1 activation was at its highest in Scenario 2. We find that this occurred roughly at hour 13, when there were 388 macrophages. We used this number of M1 macrophages as the initial conditions, along with the same amount of anti-inflammatory mediator as in Scenario 3. The legend in Fig 10 shows “ODE” and “ODE (+ receptor-bound).” In scenarios 2 and 3, this represented adding together the populations bound and unbound to the receptor within the same simulation, whereas in this scenario, the simulations themselves are unique. The former represents the simulation where only extracellular TNF*α* is set to zero initially, whereas the latter represents the simulation where both extracellular and receptor-bound TNF*α* are set to zero. Fig 10 shows ABM and both ODE model results for average activation and extracellular mediators.

The behavior of both models is similar for M1 activation and PIM. M1 activation and PIM in both models is decreased at similar rates, although M1 resolves faster in the ODE. On the other hand, we observe significant differences in dynamics of the anti-inflammatory outputs. Max M2 activation in the ODE occurs with the initial bolus and decreases for the rest of the simulation, whereas in the ABM it decreases initially but rebounds strongly before resolution. AIM in the ODE follow M2 activation dynamics fairly closely, whereas AIM in the ABM steadily decreases from the initial value. These differences are likely due to the unique implementation of anti-inflammatory feedback loops in each model type and how activation and pro-inflammatories are defined in each model.

### Scenario 5: Half M1 and half M2

We then observed the results of initializing the models to a state of high activation such that half of the macrophages present were activated to an M1 phenotype and half were M2, with no external stimuli.

For the ODEs, we used the same initial conditions for M1 macrophages as in Scenario 4, and used a similar method to obtain initial conditions for M2. Hour 10 in Scenario 1 for both models was the time around which peak M2 activation occurs. Five macrophages were defined to have M1 initial conditions and the other five had M2 initial conditions. When receptor-bound mediators were not taken into account, only extracellular TNF*α* and IL-10 were set to zero at the beginning of the simulation. When receptor-bound mediators were considered part of the overall TNF*α* and IL-10 concentrations, they were also set to zero.

**Fig 11:**
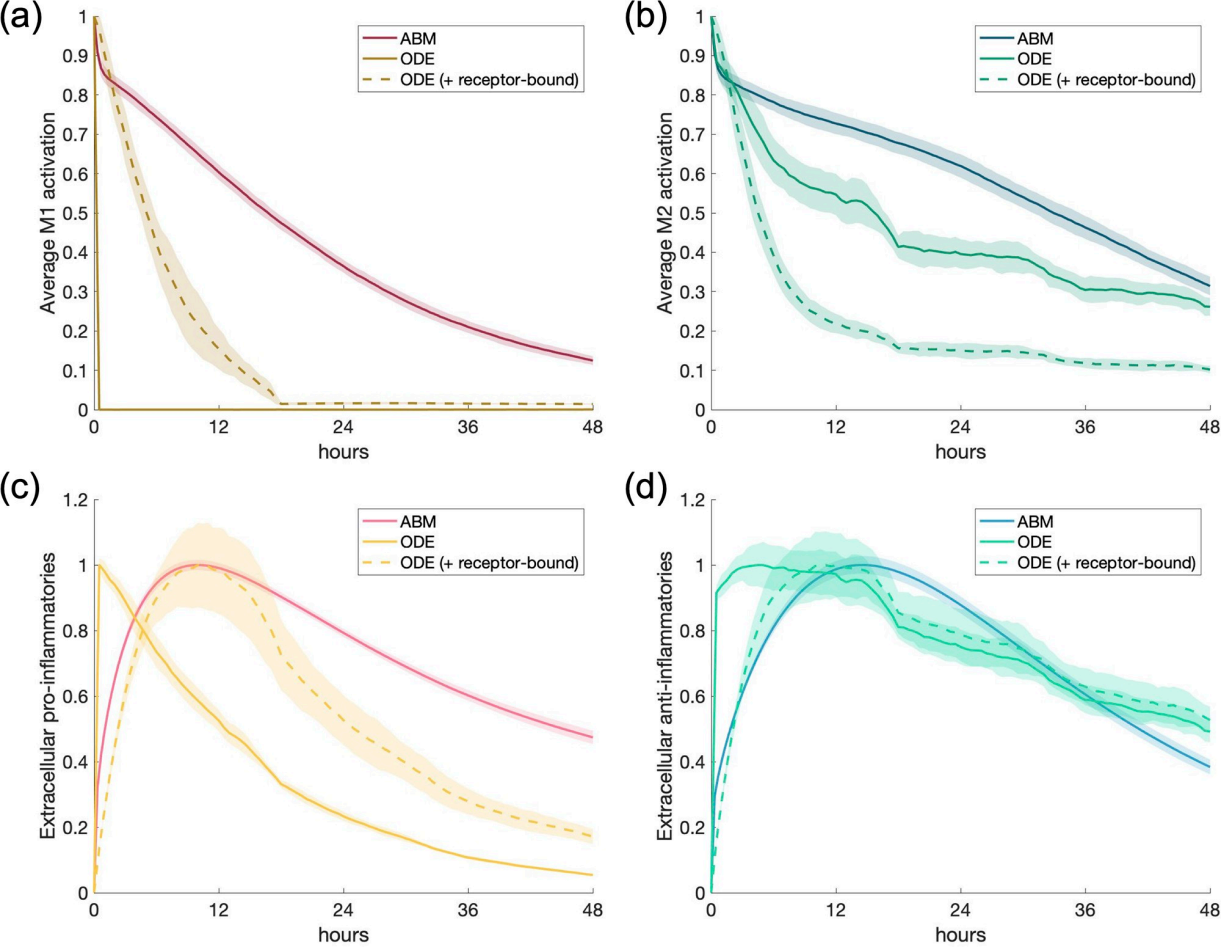
Multi-cell response to mixed M1/M2 environment. Scenario 5: M1/M2 response to a state of activation in which half of the macrophages present are M1 macrophages and half are M2. Transients are scaled individually by their maximums. “ODE (+ receptor-bound)” is the sum of the extracellular cytokine plus the receptor-bound cytokine. (a) M1 activation, (b) M2 activation, (c) PIM, (d) AIM.

For the ABM, we used a total of 388 macrophages to represent a state of high activation, similar to the maximum amount of macrophages in Scenario 4. Half were defined as M1 and half as M2. Fig 11 shows the results for M1 and M2 activation and for pro- and anti-inflammatory mediators. AIM and PIM were set to zero.

Overall behavior of all four outputs are in good agreement between the model types. The ODE simulation that includes receptor-bound mediators matches the ABM better for all outputs except M2 activation, where activation decreases faster, possibly due to longer M1 activation. Peak PIM and AIM line up well between the two models in the receptor-bound case. Furthermore, in this scenario, the ABM model generates longer tails in activation and PIM than in the ODE. This may be the result of a larger number of macrophages with longer lifespans in the ABM as compared to the setup of the ODE model.

### Scenario 6: PIM activation with wash and anti-inflammatory stimulus

**Fig 12:**
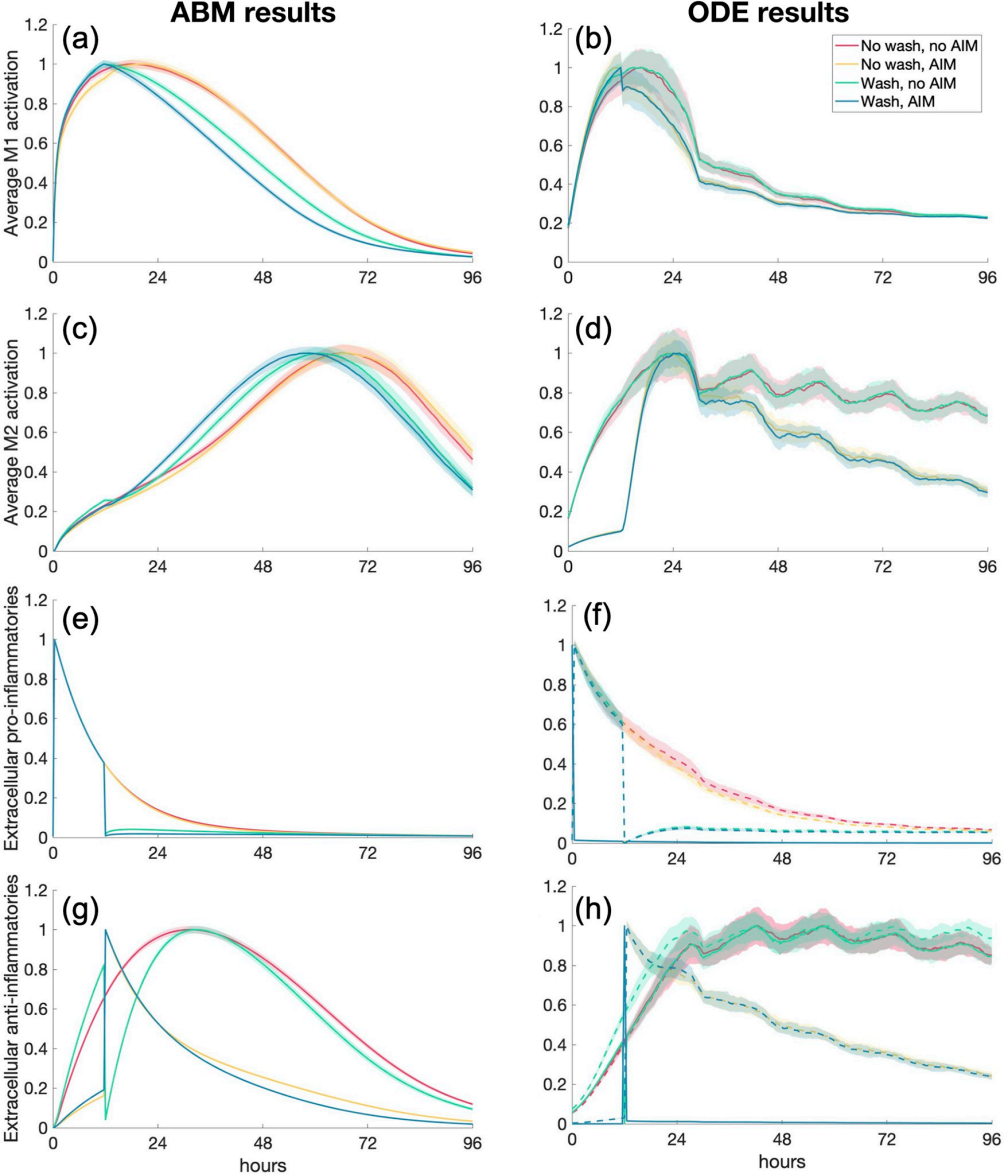
*In silico* simulation of a wash experiment. Scenario 6: M1/M2 response to an initial pro-inflammatory stimulus and either wash or no wash, with AIM added or not added at hour 12. Transients are scaled individually by their maximums. “ODE (+ receptor-bound)” is the sum of the extracellular cytokine plus the receptor-bound cytokine. Column 1: ABM results. Column 2: ODE results. (a, b) M1 activation, (c, d) M2 activation, (e, f) PIM, (g, h) AIM.

It is common in experimental setups to perform a wash, where cells are treated with a stimulus, then “washed” with a solution to remove external mediators [32]. We replicated this experiment by beginning with the same initial conditions as in Scenario 2: 10 naive macrophages and a pro-inflammatory stimulus (30 for the ABM. Then at hour 12, the cells, at whatever state they were in at that time, were “washed” such that PIM and AIM were set to zero and a high amount of AIM was added (same as initial amount in Scenario 3). In the case of considering receptor-bound mediators, receptor-bound TNF*α* was also set to zero at hour 12. Results are shown in Fig 12. Since the times at which M1 and M2 activation is affected most by the wash is different for the ABM versus the ODE model, we compare experiments to examine how they differ from a control, where there is no wash and no AIM added at 12 hours. Therefore, we show four cases, all of which are initialized with PIM: 1) PIM with no later intervention, 2) no wash, AIM added at 12 hours, 3) wash with AIM added, 4) wash with no AIM added. In the future, experiments could be performed with data collected when the models’ dynamics differ significantly in order to select which model best replicates the experimental results.

Fig 12(a)-12(d) shows that M1 and M2 activation in both models responds similarly regardless of the experiment. ABM transients look almost identical across all four simulations while M2 activation in the ODE shows a slightly faster rate of decrease for the experiments with an AIM bolus at hour 12 than that with no bolus. The ODE model has a more immediate response to the AIM than the wash, shown in the sharp changes at hour 12 for the blue and yellow curves in panels (b) and (d). On the other hand, activation in the ABM does not show these sharp changes; rather, they are more gradual even though large jumps are reflected in the PIM and AIM dynamics. Incorporating the receptor-bound mediators into the extracellular AIM and PIM in the ODE simulations shows a good match with the ABM results in panels (e) through (h). Similarly to the calibrating experiment, ODE transients have longer tails than those from the ABM—especially M2 activation and anti-inflammatories. Furthermore, in both model types, M1 activation generally peaks before M2 activation although M2 peaks much later in the ABM than in the ODE. Examining these four scenarios allowed us to observe how the models respond to different variations of stimuli and pinpoint the sensitivity of both models to these stimuli. It could also aid in selecting the best way to create an *in silico* representation of an experiment such as a wash.

### Sensitivity analysis results

**Fig 13:**
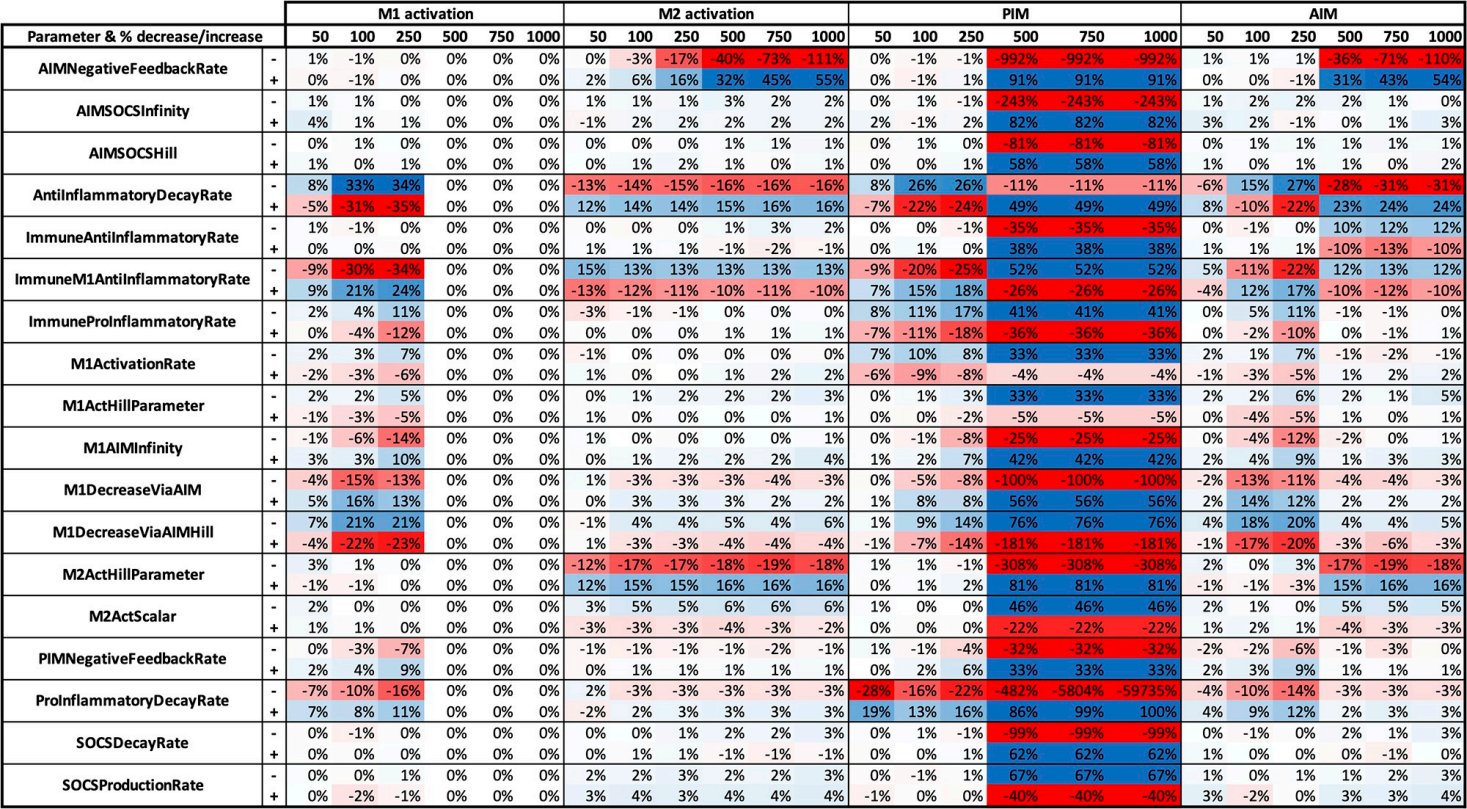
Sensitivity analysis results for single-macrophage ODE model parameters. Parameters were perturbed by increasing or decreasing the original value by 10%, shown as “+” and “-”, respectively, next to each parameter name. Parameters with 20% change or more for at least one variable and time point are shown. Red and blue boxes represent a decrease and increase, respectively, in the value from baseline. The darker the color, the greater the change from baseline.

**Fig 14:**
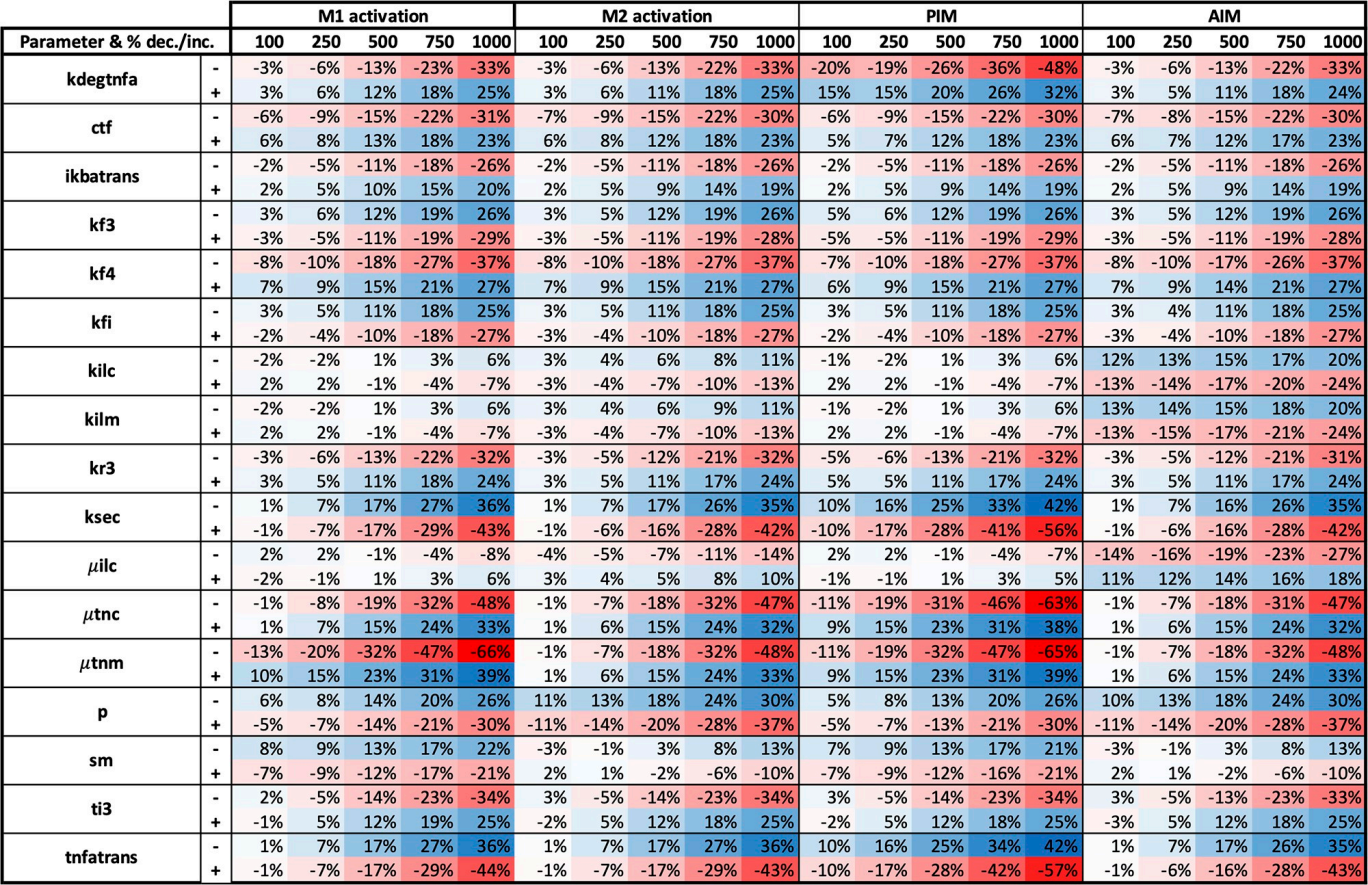
Sensitivity analysis results for single-macrophage ABM parameters. Parameters were perturbed by increasing or decreasing the original value by 10%, shown as “+” and “-”, respectively, next to each parameter name. Parameters with 30% change or more for at least one variable and time point are shown. Red and blue boxes represent a decrease and increase, respectively, in the value from baseline. The darker the color, the greater the change from baseline.

**Fig 15:**
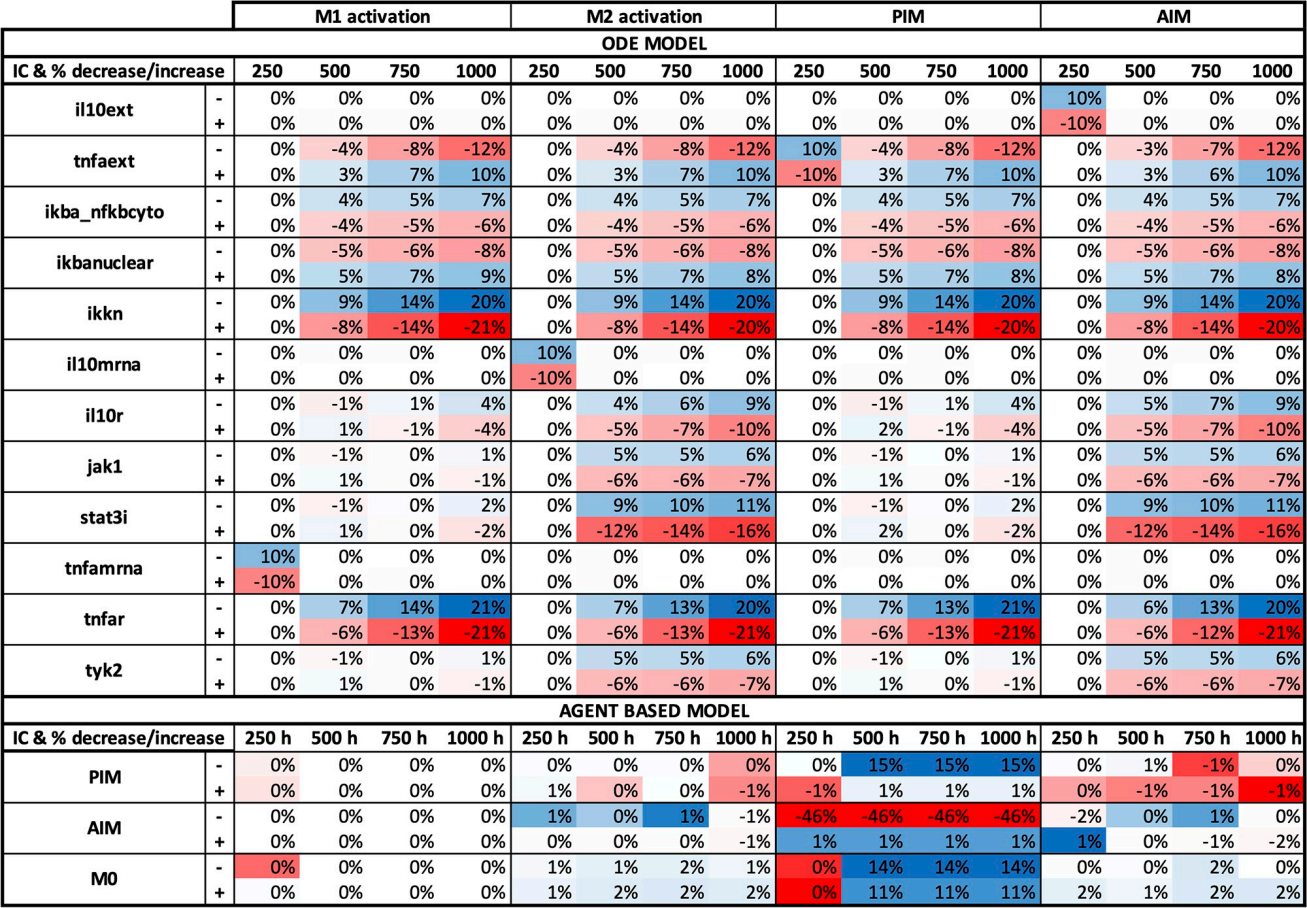
Sensitivity analysis results for single-macrophage initial conditions (ODE and ABM). Initial conditions were perturbed by increasing or decreasing the original value by 10%, shown as “+” and “-”, respectively, next to each variable name. Variables with a nonzero change for at least one variable and time point are shown. Red and blue boxes represent a decrease and increase, respectively, in the value from baseline. The darker the color, the greater the change from baseline.

Through performing a sensitivity analysis, we were able to determine model parameters and initial conditions to which M1/M2 activation and concentrations of PIM and AIM are most sensitive. We also compared the results between the two models and distinguish whether these key parameters are model-specific or common to both. Parameter results for the single-macrophage ODE and ABM model are shown in Figs 13 and 14, respectively. Time points that showed little sensitivity for all outputs are not shown in the sensitivity analysis result figures. Furthermore, we only show results for parameters with a percent change greater than 30% for at least one variable and time point. We also show sensitivity analysis results for initial conditions of the ODE model and ABM. Variables with a nonzero change for at least one variable and time point are shown in Fig 15.

Of the 56 parameters in the ODE model, 17 show changes by more than 20% for at least one time point and variable tested, shown in Fig 13. The majority of these sensitive parameters are found in TNF*α*-related equations, relating to its activation and transcription, such as *μ*_*tnc*_, *μ*_*tnm*_, *k*_*sec*_, and *k*_*tnfatrans*_. A number of other highly sensitive parameters are found in equations involving IL-10 production and transport outside the cell. A few parameters relating to the signaling pathways involving I*κ*B*α* and NF*κ*B were sensitive, pointing to the possibility that these steps may not need to be modeled explicitly.

In the ABM, 18 parameters result in changes by more than 30% for at least one variable and one time point, shown in Fig 14. M1 activation is very sensitive to many of the parameters at early time points and not sensitive at all for the later time points. This is due to the shorter time course of M1 activation in the ABM, where M1 activation returns to 0 within about 250 hours and does not increase again, in contrast to the ODE model where M1 activation decreases more gradually after the peak. Furthermore, the 0% sensitivity after 250 hours in the ABM highlights the discrete nature of species in this type of model; once M1 macrophages disappear from the grid, they do not return. In contrast, PIM, a continuous variable that diffuses throughout the grid, shows high sensitivity at all time points measured; however, this is more so due to the nearly negligible amounts of PIM at later time points, resulting in seemingly high sensitivity. This highlights how the type of model can affect sensitivity results. Furthermore, in contrast to the ODE model, the highly sensitive parameters found from the ABM do not focus on any particular mechanisms; rather, they reflect a variety of steps in the immune response, from M1 and M2 activation (M1ActivationRate, M2ActHillParameter, M2ActScalar) to SOCS activation and regulation SOCS (AIMSOCSInfinity, M2SOCSInfinity, SOCSProductionRate). The nonlinear relationships used are important to the model, shown in the sensitivity of parameters such as M2ActHillParameter.

Initial conditions in the ODE model are not extremely sensitive. Changes in ICs within 10% reveal result in output changes less than 25%. Fig 15 shows the 12 variables with the greatest variation. Sensitive state variables are spread fairly evenly through both the pro- and anti-inflammatory pathways. Time points after 250 hours are typically more sensitive. Variables related to the pro-inflammatory pathway such as *TNFα*_*ext*_, *IκBαNFκB*_*cyto*_, and *IKK*_*n*_ are sensitive for all outputs whereas those related to the anti-inflammatory pathways such as *IL*10*R*, *Jak*1, and *STAT*3_*i*_ show sensitivity only for M2 and AIM. For the agent-based model, PIM is quite sensitive to all three agents scanned, while the other outputs of interest are quite insensitive.

## Discussion

With still much unknown about M1-M2 polarization and the important role it plays in the pathogenesis of many diseases [5], our modeling approaches and scenarios shed light on the biological mechanisms, modeling approaches, and areas of uncertaintly on which to focus future efforts in understanding macrophage polarization. Our comparison of *in silico* platforms provides the ability to test hypotheses and highlight mechanisms that may be necessary or unnecessary to include in future models.

By using the same basic principles of M1/M2 activation, interaction with mediators, and cell lifespan, our two distinctly different models provided surprisingly similar results after tuning to a common calibrating experiment. In particular, peak times and overall shapes of the transients were similar in most cases. Whereas our ODE model accounted for relatively detailed subcellular signaling, where each term represented a different interaction within the cell as well as with extracellular mediators, our ABM simplified the interactions to reflect similar roles of M1/M2 activation without the detail of individual mechanisms and interactions. Rather, only M1/M2 activation and mediators were measured in the model.

A common difference between models was a longer tail of M2 activation and AIM activity across several scenarios. This was also seen in the calibrating scenario, where M2 activation decreases more quickly in the ABM. Future work could include finer tuning of the parameters to better align the model results and understand the exact mechanisms driving this difference.

Another thread throughout this work is the consideration of receptor-bound TNF*α* and IL-10 in the ODE model. Through simulating the calibrating experiment and scenarios, described below, we found that receptor-bound TNF*α* and IL-10 in the ODE model played an important role in the resulting dynamics. It is often assumed that changes in cytokine levels due to receptor binding is negligible. However, we found that this is not the case in our ODE model likely due to its small-scale nature, and explicitly modeling receptors makes a difference in dynamics. In most scenarios, especially those with high amounts of one mediator (Scenarios 2–4) or both (Scenarios 5–6), incorporating receptor-bound mediators into the overall concentration of mediators improved similarity to the ABM results. Though this disparity was initially unexpected, it was not surprising since the ABM does not explicitly model receptors such that extracellular mediators are not removed from the population when they interact with a macrophage. Due to the significant difference when taking into account receptors versus not taking them into account, future changes to the ABM may involve accounting for receptor-bound mediators by explicitly including receptors or PIM and AIM in the extracellular population could be decreased when they come into contact with a macrophage, representing binding to receptors.

We also wanted to examine the differences that incorporating space (ABM) or detailed subcellular signaling (ODEs) would make in the resulting dynamics. A notable difference between the two models is seen in Fig 10(a), where residual amounts of M1-related variables such as intracellular forms of NF*κ*B and TNF*α* resulted in a small downstream bump in M1 activation in the ODE, whereas the ABM, which does not account for these variables, showed a more gradual, constant decrease of M1 activation to zero. Another significant difference was observed in the “wash” experience in Scenario 6, where the ODE model had a greater sensitivity to immediate changes in PIM and AIM than the ABM.

In the ABM, rules of macrophage activation are defined such that activation decreases gradually when a stimulus is not present, whereas in the ODE model the explicit transcription of mRNA responds directly and more immediately to a lack of extracellular mediators. Interestingly, this discrepancy did not significantly affect the other scenarios. This is an area of future investigation, especially if these models could be validated with experimental data. Overall, the incorporation of multi-step subcellular signaling was not very important since the ABM did not include subcellular signaling and we obtained very similar dynamics from both models.

We did not observe significant differences regarding the spatial dynamics of the ABM versus the well-mixed assumption of the ODE model, although this was not a focus of our analysis. It has been shown in previous ABMs involving macrophages, such as modeling granuloma formation in tuberculosis [33], that incorporating the ability of macrophages to interact on a spatial level and gather together is important to the immune response. Future simulations and scenarios could involve putting initial amounts of PIM and AIM on different areas within the grid or in different patterns to observe more carefully how space plays a role in M1/M2 activation.

Our sensitivity analysis showed that in the ODE model, M1 & M2 activation, PIM, and AIM were not highly sensitive to parameters relating to the NF-*κ*B pathway. This suggests that these mechanisms may not need to be modeled explicitly. In the ABM, the mechanisms reflected in the highly sensitive parameters were more evenly distributed among each step of the series of rules, suggesting that the mechanisms included were each necessary to capture the dynamics of the variables of interest. Additional understanding of uncertainty in the two models could be ascertained by examining how sensitivity differs between scenarios, and how the discrete aspects of the ABM contribute to the sensitivity profiles of the variables of interest.

Based on our findings from comparing the two models, we recommend a focus on the main interactions of extracellular mediators and macrophages, where M1/M2 polarization can occur on a continuous spectrum, reflecting the current knowledge and modeling practices of macrophage activation [9, 34, 35]. Important feedback loops in the pro- and anti-inflammatory phases of the immune response are: the positive feedback loop of M1 activation, upregulation of M2 via M1, and the negative feedback loop in which M2 decreases both M1 and itself. Initially, our ABM did not include SOCS, a family of intracellular proteins produced by the IL-10 pathway to regulate itself. Without this regulatory feedback loop, M2 activation and AIM did not decrease back to its initial state, but when we added SOCS to the ABM, we obtained the expected dynamics such that the calibrating experiment results of the ABM were similar to the ODE model, which did include SOCS. Whether these interactions and feedback loops are modeled explicitly through signaling pathways or through general rules was less important for our purposes, as our results from the two approaches were similar, as long as they were included in some manner.

Future work necessary to confirm our hypotheses via the scenarios described above is to fit both models, especially the calibrating experiment, to additional data. Thus far, the ODE model based on LPS-induced dynamics utilized data from Maiti et al. to tune the parameters. More sophisticated parameter estimation methods, such as obtaining correlations between parameters and a sensitivity analysis, would be useful due to the large number of parameters in the model, and could potentially reduce model complexity. Parameter estimation and selection of parameter values and cell lifespans can be updated to reflect the different timescales of cell death, molecular binding, and cellular activation. In addition, these models represent a localized response to observe how macrophages in close proximity may interact with their surrounding pro- or anti-inflammatory environment. These local models can be coupled to represent a larger space, and parameters and initial conditions can be chosen to reflect different regions or conditions. Furthermore, currently both models are meant to represent the immune response to a general insult. These models can be adapted to incorporate the key players and mechanisms involved in specific injuries such as bacterial or viral infections, wound healing, sepsis, or COPD.

## Supporting information

S1 File (PDF)

S1 Text (M)

S2 Text (M)

S3 Text (M)

S4 Text (M)

S5 Text (M)

S6 Text (M)

S7 Text (M)

S8 Text (M)

S9 Text (M)

S10 Text (M)

S11 Text (M)
