# Supplementary material for: Agent-based vs. equation-based multi-scale modeling for macrophage polarization
Source: PLoS One. 2024 Jan 25;19(1):e0270779. doi: 10.1371/journal.pone.0270779 (PMC10810539; doi:10.1371/journal.pone.0270779)
Supplement: S1 File — (PDF) [file pone.0270779.s001.pdf]

Agent-based vs. equation-based multi-scale modeling for macrophage polarization
Supplemental Materials

Sarah Minucci, Rebecca Heise, Angela Reynolds

Model equations

The full model is represented by Equations (1) through (35). We have noted in the text where our model differs from that of Maiti et al. [2]. Maiti et al. began the model through initiation by LPS, a major component of bacteria identified by the macrophage. Since this model replicates a sterile injury, we removed LPS and its related equations and instead used TNFα as the initiator of the signaling cascade. Furthermore, we continue with the convention established by Maiti et al. in that the total number of proteins maintained by the cell surface remains roughly the same at baseline. Therefore we do not include synthesis and elimination terms for state variables representing receptors, whereas synthesis and elimination terms are included for the other components of the cascade.

IκBα kinase

IκBα kinase (IKK) is represented in three distinct states: neutral, active, and inactive, shown in Eqs (1), (2), and (3), respectively. As part of a negative feedback loop for the pro-inflammatory response, IL-10 inhibits neutral IKK from activating. Maiti et al. describes this inhibition in the first term of Eqs (1) and (2) through the parameter k_{in}, where

$$k_{in} = \max\left(1 - \frac{IL10/R}{IL10/R_{max}}, 0\right).$$

Active IKK phosphorylates the IKK-IκBα-NFκB complex (second term in Eq (2)). Phosphorylation causes the complex to break down, releasing a neutral form of IKK, shown in the second term of Eq (1). Finally as part a negative feedback loop to prevent an overactive pro-inflammatory response, the protein A20 inactivates active IKK, the last term of Eq (2) and Eq (3).

$$\frac{dIKK_n}{dt} = -\overbrace{k_{fi}k_{in}TNF\alpha/R\ IKK_n}^{\text{IKK activation}} + \overbrace{t_{i3}IKK/I\kappa B\alpha/NF\kappa B_{cyto}}^{\text{Complex breaks down}} \tag{1}$$

$$\begin{aligned}\frac{dIKK_a}{dt} =& \overbrace{k_{fi}k_{in}TNF\alpha/R\ IKK_n}^{\text{IKK activation}} - \overbrace{k_{k3}k_{in}IKK_a I\kappa B\alpha/NF\kappa B_{cyto}}^{\text{IKK binds to I}\kappa\text{ B}\alpha/\text{NF}\kappa\text{B}} \\ & - \overbrace{k_{k1}IKK_a A20_{cyto}}^{\text{A20 deactivates IKK}}\end{aligned} \tag{2}$$

$$\frac{dIKK_i}{dt} = \overbrace{k_{k1}IKK_a A20_{cyto}}^{\text{A20 deactivates IKK}} \tag{3}$$

IκBα

In a resting state, IκBα sequesters free NFκB by associating into a complex, shown in the first term of Eq (4). This process also occurs in the nucleus, from which the complex can move to the cytosol (second term of Eq (4)). Activated IKK phosphorylates the complex, represented by the third term in Eq (4). The binding of active IKK to IκBα-NFκB (first term of Eq (5)) causes all three components to separate, modeled by the second term of Eq (5): NFκB is released, IκBα is degraded, and IKK returns to a neutral state.

$$\begin{aligned}\frac{dI\kappa B\alpha/NF\kappa B_{cyto}}{dt} =& \overbrace{k_{f4}NF\kappa B_{cyto}I\kappa B\alpha_{cyto}}^{\text{Association}} + \overbrace{e_{ni}I\kappa B\alpha/NF\kappa B_{nuclear}k_v}^{\text{Moves outside nucleus}} \\ & - \overbrace{k_{k3}k_{in}IKK_aI\kappa B\alpha/NF\kappa B_{cyto}}^{\text{IKK binds to I}\kappa\text{B}\alpha/\text{NF}\kappa\text{B}} \qquad (4)\end{aligned}$$

$$\frac{dIKK_a/I\kappa B\alpha/NF\kappa B_{cyto}}{dt} = \overbrace{k_{k3}k_{in}IKK_aI\kappa B\alpha/NF\kappa B_{cyto}}^{\text{IKK binds to I}\kappa\text{B}\alpha/\text{NF}\kappa\text{B}} - \overbrace{t_{i3}IKK/I\kappa B\alpha/NF\kappa B_{cyto}}^{\text{Complex breaks down}} \qquad (5)$$

Eqs (6) through (9) show the various states of the inhibitory protein IκBα. NFκB promotes the transcription of IκBα mRNA, shown in the first term of Eq (6). Subsequent translation of the protein and decay of the mRNA are described in the first term of Eq (7) and the second term of Eq (6), respectively. As previously described, the second term of Eq (7) represents IκBα sequestering free NFκB in the cytosol. In a resting cell, excess IκBα is distributed evenly between the cytosol and nucleus; thus, the last two terms of Eq (7) show import and export of IκBα between the two compartments [1]. The parameter k_v accounts for the nuclear-cytoplasmic ratio to account for the size of the cell's cytoplasm in relation to its nucleus. The release of NF-κB from the IκBα-NF-κB complex by active IKK results in the phosphorylation of IκBα and its subsequent degradation, shown in the two terms of Eq (9).

$$\frac{dI\kappa B\alpha_{mrna}}{dt} = \overbrace{\frac{s_m p NF\kappa B_{nuclear}}{c_{tf} + NF\kappa B_{nuclear}}}^{\text{Transcription via NF}\kappa\text{B}} - \overbrace{\mu_{ilm}I\kappa B\alpha_{mrna}}^{\text{Decay}} \qquad (6)$$

$$\begin{aligned}\frac{dI\kappa B\alpha_{cyto}}{dt} =& \overbrace{k_{ikbatrans}I\kappa B\alpha_{mrna}}^{\text{Translation}} - \overbrace{k_{f4}NF\kappa B_{cyto}I\kappa B\alpha_{cyto}}^{\text{Association}} \\ & - \overbrace{i_{ki}I\kappa B\alpha_{cyto}}^{\text{Import to nucleus}} + \overbrace{e_{ki}I\kappa B\alpha_{nuclear}k_v}^{\text{Export from nucleus}} \qquad (7)\end{aligned}$$

$$\frac{dI\kappa B\alpha_{nuclear}}{dt} = -\overbrace{k_{f4}NF\kappa B_{nuclear}I\kappa B\alpha_{nuclear}}^{\text{Association}} + \overbrace{\frac{i_{ki}}{k_v}I\kappa B\alpha_{cyto}}^{\text{Import to nucleus}} - \overbrace{e_{ki}I\kappa B\alpha_{nuclear}}^{\text{Export from nucleus}} \qquad (8)$$

$$\frac{dI\kappa B\alpha_{phospho}}{dt} = \overbrace{t_{i3}IKK_a/I\kappa B\alpha/NF\kappa B_{cyto}}^{\text{IKK releases NF}\kappa\text{B}} - \overbrace{k_{degikba}I\kappa B\alpha_{phospho}}^{\text{Decay}} \qquad (9)$$

NFκB

The protein NFκB is released from the complex (first term of Eq (10)) and translocates to the nucleus, represented by the second term of Eq (10) [1]. NFκB activates the transcription of several genes, including TNFα and IL-10, A20, and IκBα. IκBα sequesters nuclear NFκB (last term in Eq (11) and first term in Eq (12)) before the complex moves back into the cytosol, shown in the last term of Eq (12).

$$\frac{dNF\kappa B_{cyto}}{dt} = \overbrace{t_{i3}IKK_a/I\kappa B\alpha/NF\kappa B_{cyto}}^{\text{IKK releases NF}\kappa\text{B}} - \overbrace{i_{ln}k_{in}NF\kappa B_{cyto}}^{\text{Moves to nucleus}} - \overbrace{k_{f4}NF\kappa B_{cyto}\ I\kappa B\alpha_{cyto}}^{\text{I}\kappa\text{B}\alpha\text{ sequesters NF}\kappa\text{B}} \tag{10}$$

$$\frac{dNF\kappa B_{nuclear}}{dt} = \frac{\overbrace{i_{ln}k_{in}NF\kappa B_{cyto}}^{\text{Moves to nucleus}}}{k_v} - \overbrace{k_{f4}NF\kappa B_{nuclear}I\kappa B\alpha_{nuclear}}^{\text{I}\kappa\text{B}\alpha\text{ sequesters NF}\kappa\text{B}} \tag{11}$$

$$\frac{dI\kappa B\alpha/NF\kappa B_{nuclear}}{dt} = \overbrace{k_{f4}NF\kappa B_{nuclear}I\kappa B\alpha_{nuclear}}^{\text{I}\kappa\text{B}\alpha\text{ sequesters NF}\kappa\text{B}} - \overbrace{e_{ni}I\kappa B\alpha/NF\kappa B_{nuclear}}^{\text{Moves outside nucleus}} \tag{12}$$

TNFα

One of the main targets of gene expression of NFκB is the pro-inflammatory cytokine TNFα. The first term of Eq (13) represents transcription of mRNA. There is evidence that Suppressor of Cytokine Signaling 3 (SOCS3), discussed in further detail below, plays a role in regulating the pro-inflammatory response by inhibiting TNFα mRNA and protein production, although the exact mechanisms by which this occurs is still unclear [3, 4]. We included a multiplier, not in the original equation by Maiti et al., in this first term to represent inhibition of mRNA production by SOCS3. After transcription and translation, TNFα is secreted from the cell (first two terms of Eq (14)). The parameter k_{bal} represents a component balance for TNFα as it moves from the cytosol (intracellular space) to the supernatant (extracellular space).

Extracellular TNFα binds to its receptor on the cell surface, represented by the second term in Eq (15). In some cases the cytokine unbinds from its receptor, accounted for by the second term in Eq (15). Once inside the cell, either after binding to its receptor or being translocated from the nucleus, TNFα performs several important roles. Shown in the first term of Eq (1), TNFα bound to its receptor upregulates activation of IKK, which then precipitates further NFκB transcription.

$$\frac{dTNF\alpha_{mrna}}{dt} = \overbrace{\frac{s_m p\ NF\kappa B_{nuclear}}{c_{tf} + NF\kappa B_{nuclear}}}^{\text{Transcription via NF}\kappa\text{B}} \overbrace{\left(\frac{1}{1+\left(\frac{SOCS3_{cyto}}{SOCS3_{\infty}}\right)^2}\right)}^{\text{Inhibition by SOCS3}} - \overbrace{\mu_{tnm}TNF\alpha_{mrna}}^{\text{Decay}} \tag{13}$$

$$\frac{dTNF\alpha_{cyto}}{dt} = \overbrace{k_{tnfatrans}TNF\alpha_{mrna}}^{\text{Translation}} - \overbrace{k_{sec}TNF\alpha_{cyto}}^{\text{Secreted from cell}} - \overbrace{\mu_{tnc}TNF\alpha_{cyto}}^{\text{Decay}} \tag{14}$$

$$\frac{dTNF\alpha_{ext}}{dt} = \overbrace{k_{sec}k_{bal}TNF\alpha_{cyto}}^{\text{Secreted from cell}} - \overbrace{k_{f3}TNF\alpha_{ext}TNF\alpha R}^{\text{TNF}\alpha\text{ binds to receptor}} + \overbrace{k_{r3}TNF\alpha/R}^{\text{TNF}\alpha\text{ unbinds from receptor}} - \overbrace{k_{degtnfa}TNF\alpha_{ext}}^{\text{Decay}} \tag{15}$$

$$\frac{dTNF\alpha R}{dt} = -\overbrace{k_{f3}TNF\alpha_{ext}TNF\alpha R}^{\text{TNF}\alpha\text{ binds to receptor}} + \overbrace{k_{r3}TNF\alpha/R}^{\text{TNF}\alpha\text{ unbinds from receptor}} \tag{16}$$

$$\frac{dTNF\alpha/R}{dt} = \overbrace{k_{f3}TNF\alpha_{ext}TNF\alpha R}^{\text{TNF}\alpha\text{ binds to receptor}} - \overbrace{k_{r3}TNF\alpha/R}^{\text{TNF}\alpha\text{ unbinds from receptor}} \tag{17}$$

A20

As mentioned previously, A20 is another NFκB-responsive gene responsible for deactivating IKK, which blocks NFκB translocation to the nucleus. Eq (18) shows transcription and subsequent degradation of A20 mRNA. Eq (19) shows translation of the protein in the cytosol, and A20 decays at rate k_{dega20}, second term in Eq (19).

$$\frac{dA20_{mrna}}{dt} = \overbrace{\frac{s_m \; p \; NF\kappa B_{nuclear}}{c_{tf} + NF\kappa B_{nuclear}}}^{\text{Transcription via NF}\kappa\text{B}} - \overbrace{\mu_{a20m} A20_{mrna}}^{\text{Degradation}} \tag{18}$$

$$\frac{dA20_{cyto}}{dt} = \overbrace{a_{trans} A20_{mrna}}^{\text{Translation}} - \overbrace{k_{dega20} A20_{cyto}}^{\text{Decay}} \tag{19}$$

IL-10

A hallmark of the anti-inflammatory response is the cytokine IL-10. Its gene is a target of NFκB transcription and is involved in the regulation of the pro-inflammatory response. Some events related to IL-10 production and function are included in the model by Maiti et al. [2], but we expand the model to include a fuller view of the role of IL-10 and an important pathway it activates.

Extracellular IL-10 can bind to and unbind from its receptor IL-10R, as modeled by the first two terms in Eq (20) [5]. For simplicity, we assume the total number of receptors is conserved. The first term in Eq (22) describes upregulation of the IL-10 gene by transcription factors NFκB and STAT3. Maiti et al. include the constants 0.4 and 0.6 such that NFκB is responsible for 40% of the transcription rate and STAT3 is responsible for the other 60%. The nonlinear terms represent maximum possible rates of IL-10 transcription, since space in the nucleus is limited. IL-10 is translated from its mRNA and secreted from the cell (first two terms of Eq (23)). The third term in Eq (20) includes a component balance k_{bal} between the cytosol and supernatant. Baseline degradation rates for extra- and intracellular IL-10 and IL-10 mRNA is included in Eqs (20), (23), and (22), respectively.

$$\begin{aligned}\frac{dIL10_{ext}}{dt} =& - \overbrace{k_{ilrb} IL10_{ext} IL10R}^{\text{Binds to receptor}} + \overbrace{k_{ilru} IL10/R}^{\text{Unbinds from receptor}} + \overbrace{k_{ilc} k_{bal} IL10_{cyto}}^{\text{Moves outside cell}} \\ & - \overbrace{\mu_{ile} IL10_{ext}}^{\text{Decay}}\end{aligned} \tag{20}$$

$$\frac{dIL10R}{dt} = - \overbrace{k_{ilrb} IL10_{ext} IL10R}^{\text{IL-10 binds to receptor}} + \overbrace{k_{ilru} IL10/R}^{\text{IL-10 unbinds from receptor}} \tag{21}$$

$$\frac{dIL10_{mRNA}}{dt} = \overbrace{0.4 k_{ilnf} p \frac{NF\kappa B}{c_{tf} + NF\kappa B} + 0.6 k_{ilsn} p \frac{STAT3_n}{c_{tfstat3} + STAT3_n}}^{\text{Gene transcription}} - \overbrace{\mu_{ilm} IL10_{mRNA}}^{\text{Decay}} \tag{22}$$

$$\frac{dIL10_{cyto}}{dt} = \overbrace{k_{ilm} IL10_{mRNA}}^{\text{Translation}} - \overbrace{k_{ilc} IL10_{cyto}}^{\text{Moves outside cell}} - \overbrace{\mu_{ilc} IL10_{cyto}}^{\text{Decay}} \tag{23}$$

JAK-STAT signaling

Aside from inhibitory functions, IL-10 signaling initiates the JAK-STAT signaling pathway, a primary mechanism through which the immune response mediates inflammation [6]. The protein tyrosine kinases JAK1 and Tyk2 are recruited to the IL-10/IL-10 receptor complex, shown in the third term of Eq (24). This creates a new complex, $IL10/R/JAK1/Tyk2$, Eq (27) [7]. The second term accounts for the possibility that the complex may break apart. JAK1 (Eq (25)) and Tyk2 (Eq (26)) concentrations are conserved, assuming

enzyme-type dynamics. In light of the many components involved in creating this complex, we explored incorporating the various combinations of the binding steps, such as the individual receptor components, each of which bind to a specific tyrosine kinase. In the end, we decided to model the recruitment of JAK1 and Tyk2 to the IL-10/IL-10 receptor complex as one step; this still captures the appropriate dynamics without adding more parameters and equations. The last two terms of Eq (24) and all of Eqs (25) through (27) are our additions to the original model by Maiti et al., with terms representing activation of STAT3 through the Jak-STAT pathway adapted from Moya et al. [5].

$$\begin{aligned}\frac{dIL10/R}{dt} &= \overbrace{k_{ilrb} IL10_{ext} IL10R}^{\text{IL-10 binds to receptor}} - \overbrace{k_{ilru} IL10/R}^{\substack{\text{IL-10 unbinds}\\ \text{from receptor}}} \\ &\quad - \overbrace{k_{iljb} IL10/R\ JAK1\ Tyk2}^{\text{Recruitment of } JAK1 \text{ and } Tyk2} + \overbrace{k_{ilju} IL10/R/JAK1/Tyk2}^{\text{Dissociation of } JAK1 \text{ and } Tyk2} \qquad (24)\end{aligned}$$

$$\frac{dJAK1}{dt} = - \overbrace{k_{iljb} IL10/R\ JAK1\ Tyk2}^{\text{Recruitment of } JAK1 \text{ and } Tyk2} + \overbrace{k_{ilju} IL10/R/JAK1/Tyk2}^{\text{Dissociation of } JAK1 \text{ and } Tyk2} \qquad (25)$$

$$\frac{dTyk2}{dt} = - \overbrace{k_{iljb} IL10/R\ JAK1\ Tyk2}^{\text{Recruitment of } JAK1 \text{ and } Tyk2} + \overbrace{k_{ilju} IL10/R/JAK1/Tyk2}^{\text{Dissociation of } JAK1 \text{ and } Tyk2} \qquad (26)$$

$$\frac{dIL10/R/JAK1/Tyk2}{dt} = \overbrace{k_{iljb} IL10/R\ JAK1\ Tyk2}^{\text{Recruitment of } JAK1 \text{ and } Tyk2} - \overbrace{k_{ilju} IL10/R/JAK1/Tyk2}^{\text{Dissociation of } JAK1 \text{ and } Tyk2)} \qquad (27)$$

The IL-10/IL-10 receptor/JAK1/Tyk2 complex serves as a temporary docking station for inactive Signal Transducer and Activator of Transcription 3 (STAT3) [8]. Upon recruitment to the complex, STAT3 is activated and undergoes homodimerization, shown in the first term of Eq (28). Maiti et al. modeled the recruitment and activation of STAT3 through binding of STAT3 to the IL-10/IL-10R complex without Jak1 and Tyk2. We also included a multiplier representing inhibition by Suppressors of Cytokine Signaling 1 and 3 (SOCS1 and SOCS3), two IL-10 responsive genes as well as the second term of Eq (30) and Eq (31) which allow for the conservation of STAT3 in the model. SOCS1 inhibits JAK1 function by binding its SH2 domain to JAK1, preventing STAT3 from docking to the IL-10 complex. SOCS3 performs a similar role but docks to the receptor; since we do not model at the level of detail of specific binding locations, we model this inhibition as having the same result, which is preventing STAT3 from activating [9, 10, 11].

STAT3

STAT3 translocates to the nucleus (second term of Eq (29)) and controls transcription of several IL-10 responsive genes. The main inhibitor of STAT3 function is PIAS3. The protein binds to activated STAT3, preventing further transcription [12]. We model this by including a deactivation term with rate k_{sni}, shown in the second term of Eq (30). Assuming enyzme-type dynamics for all states of STAT3, the transcription factor is conserved, and deactivated nuclear STAT3 returns to the cytosol in the last term of Eq (31).

$$\frac{dSTAT3_i}{dt} = -\overbrace{2k_{stat}IL10/R/JAK1/Tyk2\ STAT3_i^2}^{\text{STAT3 activation}}\overbrace{\left(\frac{1}{1+\left(\frac{SOCS1_{cyto}+SOCS3_{cyto}}{SOCS_{\infty}}\right)^2}\right)}^{\text{Inhibition by SOCS1/3}} + \overbrace{k_{snicyto}STAT3_{ni}}^{\text{Moves to cytosol}} \tag{28}$$

$$\frac{dSTAT3_a}{dt} = \overbrace{k_{stat}IL10/R/JAK1/Tyk2\ STAT3_i^2}^{\text{STAT3 activation}}\overbrace{\left(\frac{1}{1+\left(\frac{SOCS1_{cyto}+SOCS3_{cyto}}{SOCS_{\infty}}\right)^2}\right)}^{\text{Inhibition by SOCS1/3}} - \overbrace{k_{sa}STAT3_a}^{\text{Moves to nucleus}} \tag{29}$$

$$\frac{dSTAT3_n}{dt} = \overbrace{k_{sa}STAT3_a}^{\text{Moves to nucleus}} - \overbrace{k_{sni}STAT3_n}^{\text{Deactivation}} \tag{30}$$

$$\frac{dSTAT3_{ni}}{dt} = \overbrace{k_{sni}STAT3_n}^{\text{Deactivation}} - \overbrace{k_{snicyto}STAT3_{ni}}^{\text{Moves to cytosol}} \tag{31}$$

SOCS

The inclusion of SOCS, represented in Eqs (32) through (35), is also novel to our model as compared to that by Maiti et al. Suppressors of Cytokine Signaling 1 and 3 (SOCS1, SOCS3) are upregulated via STAT3 transcription and translation, first two terms of Eqs (32) and (33), respectively [13, 14]. The last terms of these two equations represent natural degradation of the mRNA.

$$\frac{dSOCS1_{mRNA}}{dt} = \overbrace{k_{s1st}STAT3_n}^{\text{Gene transcription}} - \overbrace{k_{s1}SOCS1_{mRNA}}^{\text{Translation}} - \overbrace{\mu_{s1m}SOCS1_{mRNA}}^{\text{Decay}} \tag{32}$$

$$\frac{dSOCS3_{mRNA}}{dt} = \overbrace{k_{s3st}STAT3_n}^{\text{Gene transcription}} - \overbrace{k_{s3}SOCS3_{mRNA}}^{\text{Translation}} - \overbrace{\mu_{s3m}SOCS3_{mRNA}}^{\text{Decay}} \tag{33}$$

$$\frac{dSOCS1_{cyto}}{dt} = \overbrace{k_{s1}SOCS1_{mRNA}}^{\text{Translation}} - \overbrace{\mu_{s1c}SOCS1_{cyto}}^{\text{Decay}} \tag{34}$$

$$\frac{dSOCS3_{cyto}}{dt} = \overbrace{k_{s3}SOCS3_{mRNA}}^{\text{Translation}} - \overbrace{\mu_{s3c}SOCS3_{cyto}}^{\text{Decay}} \tag{35}$$

References

[1] Lipniacki T, Paszek P, Brasier AR, Luxon B, Kimmel M. Mathematical model of NF-B regulatory module. Journal of Theoretical Biology. 2004;228(2):195–215. doi:10.1016/j.jtbi.2004.01.001.

[2] Maiti S, Dai W, Alaniz RC, Hahn J, Jayaraman A. Mathematical Modeling of Pro- and Anti-Inflammatory Signaling in Macrophages. Processes. 2014;3(1):1–18.

[3] Dimitriou ID, Clemenza L, Scotter AJ, Chen G, Guerra FM, Rottapel R. Putting out the fire: coordinated suppression of the innate and adaptive immune systems by SOCS1 and SOCS3 proteins. Immunological Reviews. 2008;224(1):265–283. doi:10.1111/j.1600-065X.2008.00659.x.

[4] Qasimi P, Ming-Lum A, Ghanipour A, Ong CJ, Cox ME, Ihle J, et al. Divergent Mechanisms Utilized by SOCS3 to Mediate Interleukin-10 Inhibition of Tumor Necrosis Factor and Nitric Oxide Production by Macrophages. Journal of Biological Chemistry. 2006;281(10):6316–6324. doi:10.1074/jbc.M508608200.

[5] Moya C, Huang Z, Cheng P, Jayaraman A, Hahn J. Investigation of IL-6 and IL-10 signalling via mathematical modelling. IET Systems Biology. 2011;5(1):15–26. doi:10.1049/iet-syb.2009.0060.

[6] Rawlings JS, Rosler KM, Harrison DA. The JAK/STAT signaling pathway. Journal of Cell Science. 2004;117(8):1281–1283. doi:10.1242/jcs.00963.

[7] Sabat R, Grutz G, Warszawska K, Kirsch S, Witte E, Wolk K, et al. Biology of interleukin-10. Cytokine & Growth Factor Reviews. 2010;21(5):331–344. doi:10.1016/j.cytogfr.2010.09.002.

[8] Riley JK, Takeda K, Akira S, Schreiber RD. Interleukin-10 Receptor Signaling through the JAK-STAT Pathway: Requirement for Two Distinct Receptor-Derived Signals for Anti-Inflammatory Action. Journal of Biological Chemistry. 1999;274(23):16513–16521. doi:10.1074/jbc.274.23.16513.

[9] Croker BA, Kiu H, Nicholson SE. SOCS regulation of the JAK/STAT signalling pathway. Seminars in Cell & Developmental Biology. 2008;19(4):414–422. doi:10.1016/j.semcdb.2008.07.010.

[10] Tamiya T, Kashiwagi I, Takahashi R, Yasukawa H, Yoshimura A. Suppressors of Cytokine Signaling (SOCS) Proteins and JAK/STAT Pathways. Arteriosclerosis, Thrombosis, and Vascular Biology. 2011;31(5):980–985. doi:10.1161/ATVBAHA.110.207464.

[11] Yasukawa H, Misawa H, Sakamoto H, Masuhara M, Sasaki A, Wakioka T, et al. The JAK binding protein JAB inhibits Janus tyrosine kinase activity through binding in the activation loop. The EMBO Journal. 1999;18(5):1309–1320. doi:10.1093/emboj/18.5.1309.

[12] Yagil Z, Nechushtan H, Kay G, Yang CM, Kemeny DM, Razin E. The enigma of the role of Protein inhibitor of Activated STAT3 (PIAS3) in the immune response. Trends in Immunology. 2010;31(5):199–204. doi:10.1016/j.it.2010.01.005.

[13] Carey AJ, Tan CK, Ulett GC. Infection-induced IL-10 and JAK-STAT. JAK-STAT. 2012;1(3):159–167. doi:10.4161/jkst.19918.

[14] Hutchins AP, Diez D, Miranda-Saavedra D. The IL-10/STAT3-mediated anti-inflammatory response: recent developments and future challenges. Briefings in Functional Genomics. 2013;12(6):489–498. doi:10.1093/bfgp/elt028.
